# Osmotic stress and vesiculation as key mechanisms controlling bacterial sensitivity and resistance to TiO_2_ nanoparticles

**DOI:** 10.1038/s42003-021-02213-y

**Published:** 2021-06-03

**Authors:** Christophe Pagnout, Angelina Razafitianamaharavo, Bénédicte Sohm, Céline Caillet, Audrey Beaussart, Eva Delatour, Isabelle Bihannic, Marc Offroy, Jérôme F. L. Duval

**Affiliations:** 1 Université de Lorraine, CNRS, LIEC, Metz, France; 2 Université de Lorraine, CNRS, LIEC, Nancy, France

**Keywords:** Biophysics, Microbiology

## Abstract

Toxicity mechanisms of metal oxide nanoparticles towards bacteria and underlying roles of membrane composition are still debated. Herein, the response of lipopolysaccharide-truncated *Escherichia coli* K12 mutants to TiO_2_ nanoparticles (TiO_2_NPs, exposure in dark) is addressed at the molecular, single cell, and population levels by transcriptomics, fluorescence assays, cell nanomechanics and electrohydrodynamics. We show that outer core-free lipopolysaccharides featuring intact inner core increase cell sensitivity to TiO_2_NPs. TiO_2_NPs operate as membrane strippers, which induce osmotic stress, inactivate cell osmoregulation and initiate lipid peroxidation, which ultimately leads to genesis of membrane vesicles. In itself, truncation of lipopolysaccharide inner core triggers membrane permeabilization/depolarization, lipid peroxidation and hypervesiculation. In turn, it favors the regulation of TiO_2_NP-mediated changes in cell Turgor stress and leads to efficient vesicle-facilitated release of damaged membrane components. Remarkably, vesicles further act as electrostatic baits for TiO_2_NPs, thereby mitigating TiO_2_NPs toxicity. Altogether, we highlight antagonistic lipopolysaccharide-dependent bacterial responses to nanoparticles and we show that the destabilized membrane can generate unexpected resistance phenotype.

## Abstract (web summary)

Pagnout et al investigate the multiscale response of deep rough *Escherichia coli* mutants to TiO_2_ nanoparticle exposure and find that TiO_2_ strips the cell membrane, which triggers osmotic and then oxidative stresses. Depending on their lipopolysaccharide surface-phenotype, the bacteria respond by releasing membrane vesicles that act as electrostatic baits for TiO_2_ nanoparticles.

## Introduction

Due to their photocatalytic properties, titanium dioxide nanoparticles (TiO_2_NPs) are among the NPs that are most produced and used in consumer products^1^. The antibacterial activity of TiO_2_NPs and of related composite nanomaterials^2^ towards microorganisms has been largely evaluated at the cell population level using a battery of dose−response relationships^3–7^. This approach, though important for toxicological risk assessment, remains insufficient on its own for addressing NP−cell interactions and the biological implications thereof at a mechanistic nanolevel^8,9^. In this regard, the development of omics^10^ and fluorescence-based bioassays^11^ has increased our understanding of the molecular processes that underpin the manifestation of adverse effects of TiO_2_NPs^4–6,12–29^ and other photocatalytic nanomaterials^4,12,23,24,28–34^ on bacteria. These advances have contributed to unraveling how cell exposure conditions and physicochemical properties of TiO_2_NPs determine the toxicity^4–6,13–29^. In particular, the generation of reactive oxygen species (ROS) by TiO_2_NP photocatalysis^12^ is commonly described as a key process that leads to cell surface alteration and cell viability loss^14–21,23–25,30^. However, the genericity of this mechanism is not supported by other reports on the harmful effects of TiO_2_NPs on bacteria in the absence of light^5,13,22,26,27^, and the lack of correlation between ROS production and the toxicity under UV illumination^29^. In addition, non-ROS-related toxicity has been reported for nanomaterials other than TiO_2_NPs but with similar photocatalytic properties such as ZnONPs^32,35^, MgONPs^31^, CeO_2_NPs^33^, and fullerenes^34^. These contrasting findings, together with the occurrence of lipid peroxidation under both light and dark conditions^5^, suggest the existence of a toxicity mechanism that involves non-photocatalytically TiO_2_NP-induced ROS^36,37^. The mode of action of TiO_2_NPs in the dark has also been shown to translate into cell osmotic stress^13^ and cell membrane stress as a consequence of the electrostatic attachment of TiO_2_NPs and mechanical membrane disruption^26^. Similarly, Leung et al.^29^ argued that the toxicity of TiO_2_NPs originates from interactions between the nanoparticles and the outer membrane proteins and/or lipopolysaccharides (LPS), resulting in mechanical disruption of the cell membrane and possible entry of the nanoparticles into the cell. However, the genericity of this mechanism has been questioned by Buchman et al^38^, who showed the absence of a mechanistic connection between the toxicity of functionalized cationic AuNPs and the extent to which they bind to LPS. The above elements highlight that a comprehensive molecular description of the processes governing TiO_2_NP toxicity to bacteria is incomplete.

Surprisingly, studies on the toxicity of metal oxide nanoparticles towards Gram-negative bacteria have neglected the possible production of membrane vesicles (MVs), despite the essential defense function they play in mitigating osmotic and oxidative stress^39,40^. In addition, whilst the toxicity of TiO_2_NPs in relation to their surface chemistry has been extensively studied^5,6,26^, the role of cell surface composition has received far less attention. Accordingly, the current work has the following objectives: (i) decipher the processes that govern the toxicity of TiO_2_NPs towards bacteria with controlled LPS surface phenotype, (ii) evaluate and inter-connect the cell responses probed at the gene, single-cell, and population scales over a broad range of TiO_2_NP concentration conditions, and (iii) identify and explain the cell resistance and sensitivity patterns. Herein, we thus analyze the modes of action of TiO_2_NPs on *Escherichia coli* deep rough mutants^41^ at the molecular, single-cell, and population levels. Exposures are performed in the dark and under hypotonic conditions that limit the initial aggregation of the TiO_2_NPs. Targeted transcriptomics and single-cell nanomechanics, assessed by multiparametric atomic force microscopy (AFM), highlight that TiO_2_NPs actuate osmotic stress as a consequence of cell surface abrasion. This effect is found to be operational even at low TiO_2_NP doses. Remarkably, dysregulated expressions of genes involved in osmotic stress tolerance are found to match non-monotonous variations in cell membrane elasticity and cell Turgor pressure with increasing TiO_2_NP concentration. Additional fluorescence-based assays consistently support the observed TiO_2_NP-mediated changes in membrane permeability and cell Turgor pressure, as well as oxidative cell damage triggered by the osmotic stress at sufficiently high TiO_2_NP concentrations. The TiO_2_NP modes of action are shown to intimately depend on the molecular composition of the LPS. In particular, TiO_2_NP-induced vesiculation is evidenced for only the most sensitive mutant that harbors an unaltered LPS inner core. Direct and indirect defense functions of secreted MVs against TiO_2_NPs are further highlighted. Overall, the results show that osmotic stress and cell vesiculation are associated with either TiO_2_NP resistance or sensitivity depending on the LPS phenotype.

## Results

### *rfaG* mutation in *E. coli* leads to hypersensitivity to TiO_2_NPs

**Fig. 1:**
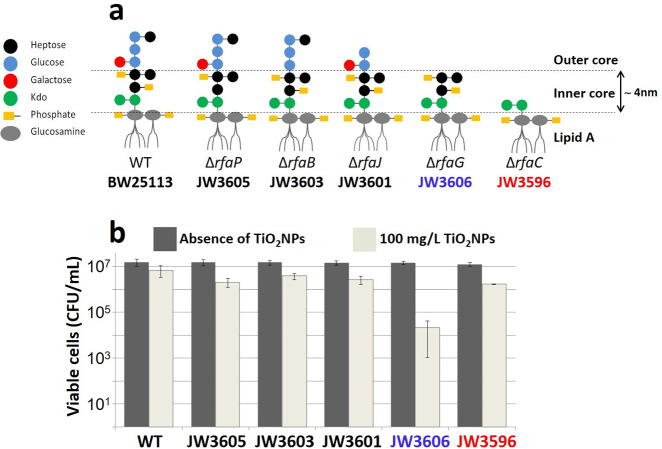
Lipopolysaccharide phenotypes and preliminary assessment of TiO_2_NP toxicity towards *E. coli* K12 *rfa-*mutants at high TiO_2_NP concentration. **a** *E. coli* K12 *rfa-*mutants, associated LPS phenotypes, and corresponding mutations in the *rfa* operon^41^. **b** Number of viable cells in CFU/mL unexposed and exposed to 100 mg/L TiO_2_NPs for 20 h in the dark. *n* = 4 for each tested condition.

**Fig. 2:**
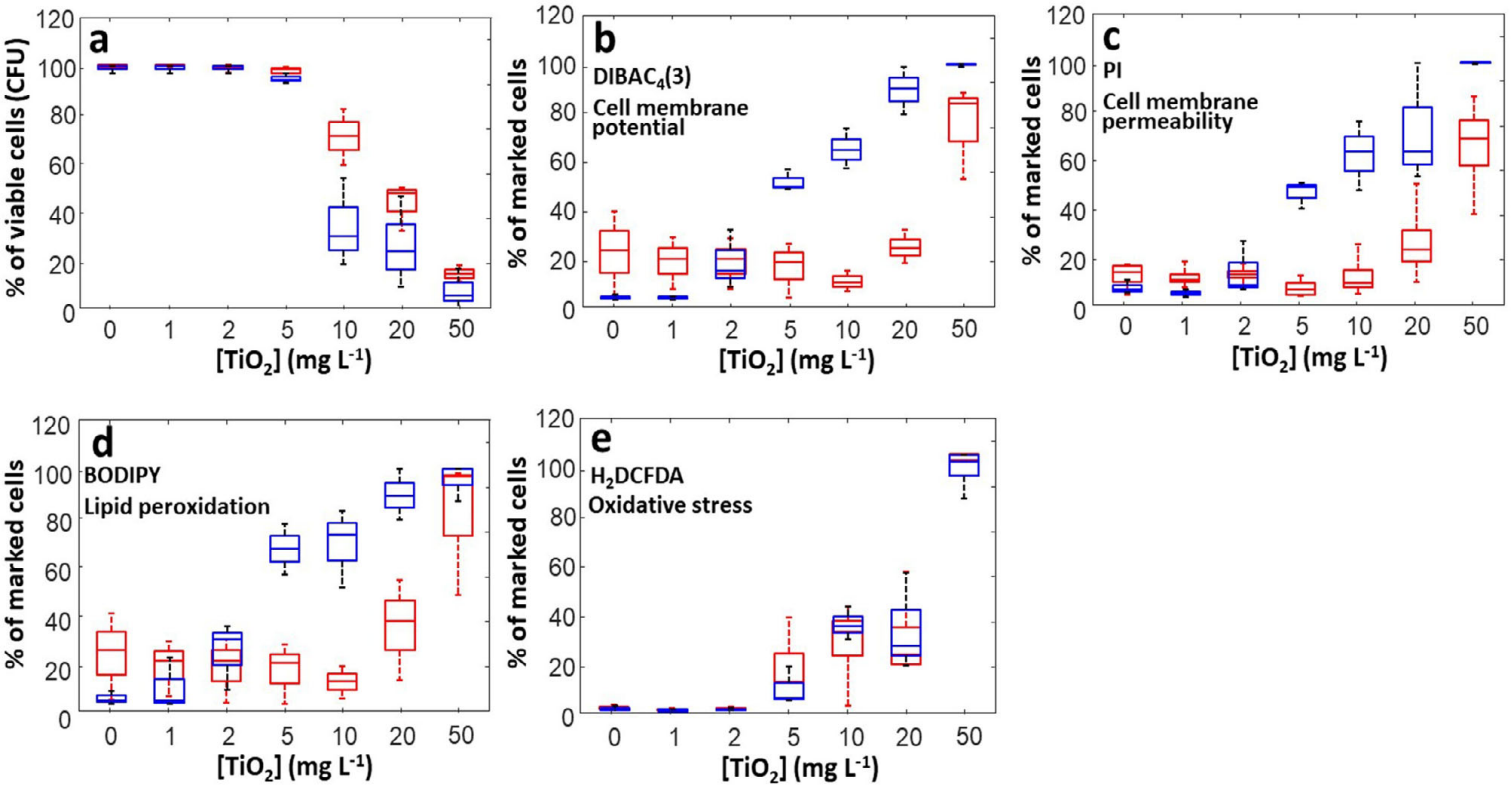
JW3606 (hep+) is the deep rough mutant that is most sensitive to TiO_2_NPs. **a** Dependence of JW3606 (hep+) (blue) and JW3596 (hep−) (red) viability on TiO_2_NP concentration. Normalized amounts of cells marked by membrane-selective fluorescent dyes for assessment of TiO_2_NP effects on **b** cell membrane potential, **c** cell membrane permeability, **d** lipid peroxidation, and **e** oxidative stress. As in (**a**), results pertaining to JW3606 (hep+) and JW3596 (hep−) are represented by blue and red boxes, respectively, in the form of box plots. Data were derived from three independent experiments for each condition examined. The selected dyes and their targeted functions are specified for each panel with DIBAC_4_(3) = bis-(1,3-dibutylbarbituric acid) trimethine oxonol, PI = propidium iodide, BODIPY = 4,4-difluoro-5,7-dimethyl-4-bora-3a,4a-diaza-s-indacene-3-propionic acid, and H_2_DCFA = 2′,7′-dichlorodihydrofluorescein diacetate. Statistical significance testing and *p*-values are provided in the Supplementary Information.

The selected *E. coli* K12 *rfa-*mutants express O-antigen-free LPS with distinct inner or outer core compositions (Fig. 1a). Of particular interest are the deep rough mutants JW3606 (Δ*rfaG*) and JW3596 (Δ*rfaC*), which lack the outer core LPS component and differ according to the presence or absence of heptose (hep) units in the inner core, respectively (Fig. 1a)^41^. In the following, JW3606 (Δ*rfaG*) and JW3596 (Δ*rfaC*) are thus referred to as JW3606 (hep+) and JW3596 (hep−). Selected TiO_2_NPs (21 nm pristine radius) display a predominantly anatase structure and are positively charged under the adopted exposure conditions (Supplementary Fig. 1a, b). Briefly, cells were exposed in the dark for 20 h to—unless otherwise specified—0–50 mg/L TiO_2_NPs at pH∼5.5–6 under agitation conditions (see “Methods”). Preliminary measurements of the colony-forming units (CFUs) on cells exposed to a high TiO_2_NP dose (100 mg/L) reveal that JW3606 (hep+) is the most sensitive to TiO_2_NPs of all *rfa*-mutants tested (∼3 log units difference, Fig. 1b). This finding underscores a connection between the toxicity of TiO_2_NPs and the LPS inner core composition. Given that the responses of the wild type (WT), JW3601 (Δ*rfaJ*), JW3603 (Δ*rfaB*), and JW3605 (Δ*rfaP*) were similar to that of JW3596 (hep−) (see Fig. 1b), the latter mutant is chosen below for a detailed comparison with JW3606 (hep+). This choice is motivated by the deep rough phenotypes of JW3596 (hep−) and JW3606 (hep+) and their comparable sensitivity to, e.g., antibiotics or detergents^42^, which strikingly contrasts with their relative sensitivity to TiO_2_NPs (Fig. 1). Figure 2a confirms the marked sensitivity of JW3606 (hep+) as compared to that of JW3596 (hep−) at TiO_2_NP concentrations higher than 5 mg/L.

Flow cytometry analysis reveals a significant impact of TiO_2_NPs on cell membrane potential (Fig. 2b), membrane permeability (Fig. 2c), and lipid peroxidation (Fig. 2d) for JW3606 (hep+) at TiO_2_NP concentrations >1 mg/L. Data further support an oxidative stress (Fig. 2e) at concentrations >2 mg/L. All of these proxies depend on the TiO_2_NP concentration according to clear dose−response relationships. The respective rates of change in lipid peroxidation (Fig. 2d) and oxidative stress levels (Fig. 2e) with increasing TiO_2_NP concentration suggest that oxidative stress alone cannot explain lipid peroxidation. Interestingly, Fig. 2 shows a marked offset between the CFU-based response of JW3606 (hep+) with increasing TiO_2_NP concentration (Fig. 2a) and that inferred from flow cytometry (Fig. 2b−e). Accordingly, provided that the TiO_2_NP concentration is ≤5 mg/L, changes in the membrane potential and permeability, lipid peroxidation level, and oxidative stress necessarily mirror the setting of cell defense mechanisms for maintaining viability (see following sections).

In agreement with Figs. 1b−2a, the situation for JW3596 (hep−) differs drastically from that for JW3606 (hep+) (Fig. 2b−e). The 10–20 mg/L TiO_2_NP concentration range marks the onset of effects on JW3596 (hep−), in contrast to the transition identified at 1–2 mg/L for JW3606 (hep+). In addition, at TiO_2_NP concentrations ≤1 mg/L and in the absence of TiO_2_NPs in the exposome, membrane depolarization, membrane permeability, and the lipid peroxidation level (Fig. 2b−d) are significantly higher for JW3596 (hep−), in line with its larger LPS inner core truncation (Fig. 1)^43^. At this stage, Fig. 2 highlights an apparent paradox: JW3596 (hep−), the mutant with a native cell membrane that is most destabilized following inner core LPS truncation (i.e., in the absence of TiO_2_NPs), is the one that exhibits greater resistance to TiO_2_NPs.

### The *ΔrfaG* mutant exhibits a TiO_2_NP-dependent vesiculation phenotype whereas hypervesiculation of the *ΔrfaC* mutant is independent of TiO_2_NP exposure conditions

**Fig. 3:**
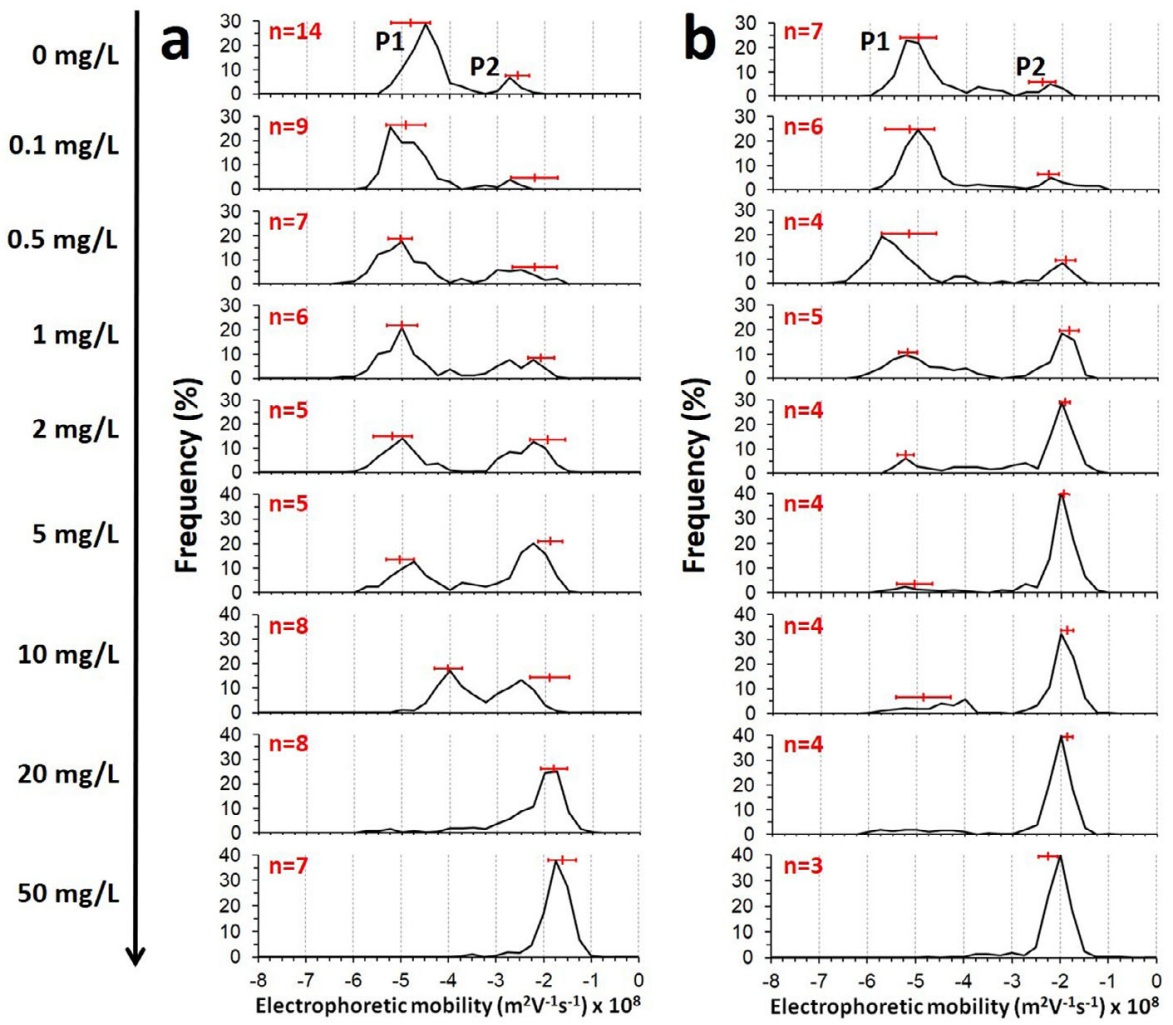
Electrokinetic fingerprints of JW3606 (hep+), JW3596 (hep−), and secreted MVs as a function of TiO_2_NP concentration. Illustrative electropherograms for (**a**) JW3606 (hep+) and (**b**) JW3596 (hep−) suspensions after 20 h incubation with TiO_2_NPs at various concentrations (indicated). Horizontal red bars correspond to the position of the maxima (mean values ± standard deviations) derived from the measurement of *n* (indicated) electrophoretic mobility distributions for the suspensions prepared from different cell cultures or colonies, with three replicates for each measurement. The reported illustrative mobility distributions are averaged over three replicates. Analysis indicates that the number of detected electrophoretic trajectories decreases by 16% and 20% in the 1–10 mg/L and 1–2 mg/L TiO_2_NP concentration regimes (analysis on 1:50 diluted JW3606 (hep+)−TiO_2_NP and JW3596 (hep−)−TiO_2_NP samples, respectively), which features significant P1 particle sedimentation under these conditions. A 61−70% increase in the number of trajectories is further observed in 1:50 diluted JW3606 (hep+)−TiO_2_NP and JW3596 (hep−)−TiO_2_NP samples, respectively, on further increasing the TiO_2_NP concentrations to 50 mg/L due to the released membrane material as a consequence of the mode of action of the TiO_2_NPs. See text for details.

Figure 3 reports the distribution profiles of electrophoretic mobilities (*µ*) for JW3606 (hep+) and JW3596 (hep−) in the presence of TiO_2_NPs (0–50 mg/L) after 20 h exposure.

Starting with the JW3606 (hep+)−TiO_2_NP system, electropherograms show the presence of two charged particle types, P1 and P2, materialized by the presence of peaks positioned at *µ*_P1_ and *µ*_P2_ in the range −4 to −5 × 10^−8^ and −1.6 to −2.6 × 10^−8^ m^2^ V^−1^ s^−1^, respectively (Fig. 3a). The apparition and extinction of the peaks depend on the TiO_2_NP concentration, which underpins variation in the number of electrophoretically detected P1 and P2 entities. P1 particles refer to JW3606 (hep+) cells as evidenced by a previous electrokinetic study performed in the absence of TiO_2_NPs^43^ and by the measurements on 0.22 µm filtered TiO_2_NP−bacteria suspensions (Supplementary Fig. 2a). The *µ*-distribution corresponding to JW3606 (hep+) is slightly shifted to negative values with increasing TiO_2_NP concentrations from 0 to 2 mg/L (Fig. 3a). With a further increase in the TiO_2_NP concentration up to 10 mg/L, the absolute value of *µ* corresponding to the maximum of the P1 (bacteria)-related peak in Fig. 3a decreases before the bacteria-associated signal completely vanishes at TiO_2_NP concentrations ≥20 mg/L. This signal suppression is due to the electrostatically favored formation of aggregates between the (negatively charged) cells (Fig. 3) and the micron-sized (positively charged) TiO_2_NP assemblies (Supplementary Fig. 1) and their subsequent sedimentation. This sedimentation process is magnified by intracellular material and cell surface residues that are possibly released under extreme stress conditions^44^. The aforementioned shift of the P1 peak to negative electrophoretic mobility values is the signature of TiO_2_NP-induced modification of the cell surface structure. Indeed, any significant adsorption of positively charged TiO_2_NPs onto JW3606 (hep+) is excluded as it should lead to a mobility shift in a direction opposite to that observed. Instead, the trend fits qualitatively with the following picture: the (negative) charges carried by the outer cell membrane surface increasingly contribute to the electrophoretic mobility of the cell via their enhanced exposure to the surrounding solution following TiO_2_NP-mediated abrasion of peripheral cell components such as LPS. This removal of protruding components of the cell surface is further accompanied by a reduction of the hydrodynamic friction exerted by the whole-cell envelope on the electroosmotic flow developed in the vicinity of the cell surface under electrophoresis conditions: such a reduction also contributes to an increase in the absolute magnitude of the cell electrophoretic mobility. These connections between the cell electrophoretic properties and the cell surface organization are in line with predictions from the soft surface electrokinetic theory^45,46^ and with previous conclusions on the impact of surface appendages on bacteria electrohydrodynamics^46,47^.

**Fig. 4:**
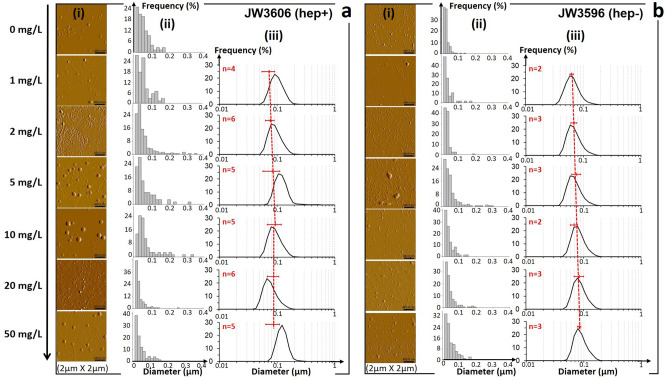
MV imaging and MV size distributions as a function of TiO_2_NP concentration. **i** AFM imaging of MVs and **ii** corresponding estimations of their size distribution, and **iii** DLS-derived MV size distribution as a function of TiO_2_NP concentration (indicated) for (**a**) JW3606 (hep+) and (**b**) JW3596 (hep-). In **a(iii)** and **b(iii)**, red dotted lines are guides to the eye and horizontal red bars correspond to the position of the maxima (mean values ± standard deviations) derived from DLS measurement of *n* (indicated) size distributions for suspensions prepared from different cell cultures or colonies, with three replicates for each measurement. The reported illustrative size distributions are averaged over three replicates. DLS measurements of MV size were performed on 0.22 μm-filtered suspensions (1:10 diluted in ultrapure water, see “Methods”) and results were confirmed by measurements on 0.45 μm-filtrated suspensions. Qualitative MV size estimations from AFM imaging were based on the analysis of 0.45 μm-filtered suspensions. Detection of MVs by DLS in the absence of TiO_2_NP was not possible due to an insufficient amount of particles in the solution.

**Fig. 5:**
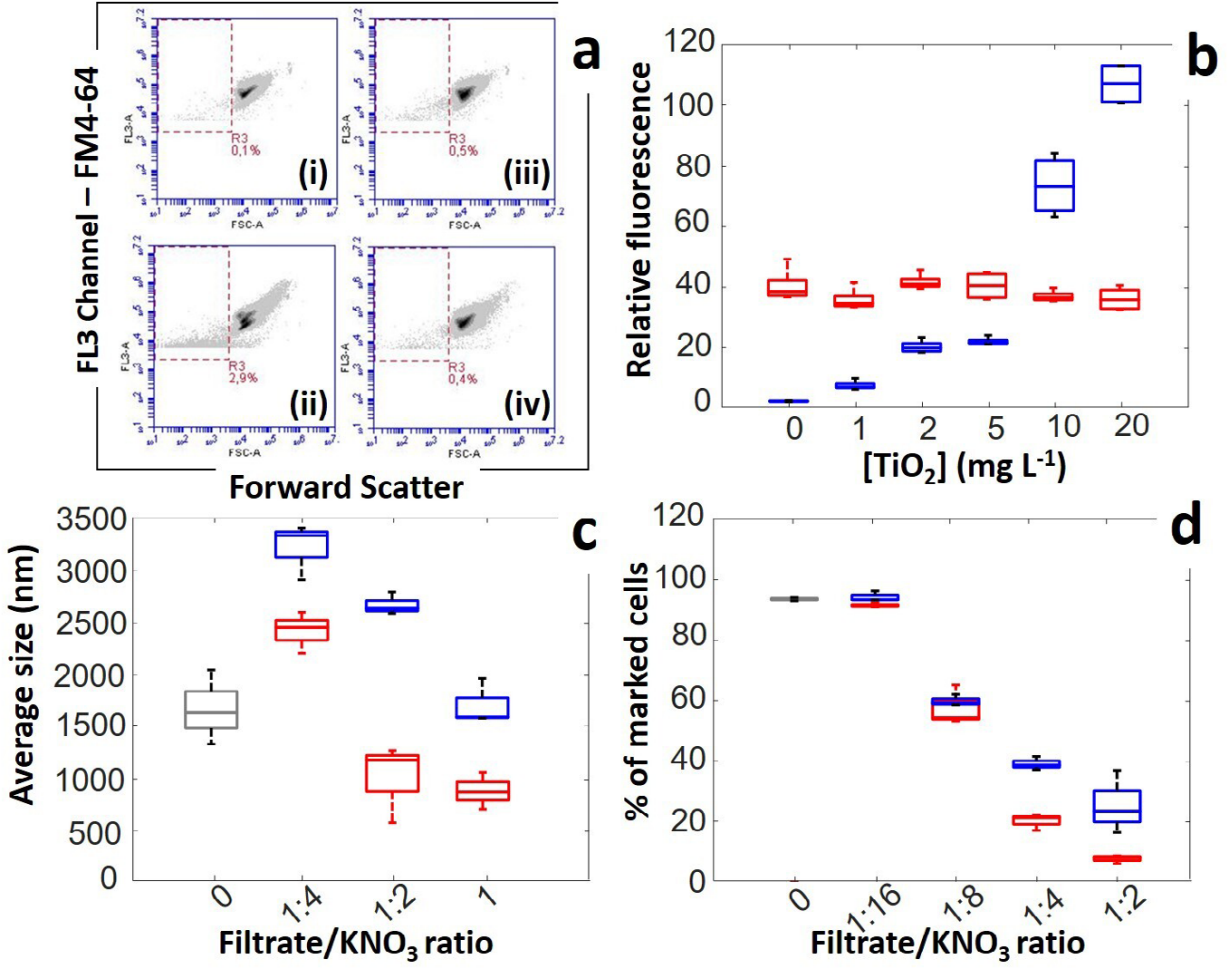
The dependences of JW3606 (hep+) and JW3596 (hep−) vesiculation phenotypes on TiO_2_NP concentration are strikingly different. **a** Illustrative cytograms with detection of FM4-64 labeled bacteria and MVs (red dotted window) (gate set on FL3 positive events) for JW3606 (hep+) unexposed **a(i)** and exposed to 20 mg/L TiO_2_NPs **a(ii)**, and JW3596 (hep−) unexposed **a(iii)** and exposed to 20 mg/L TiO_2_NPs **a(iv)**. **b** Evaluation of secreted MV amounts expressed in relative fluorescence intensity units after Syto9 labeling as a function of TiO_2_NP concentration (see *Methods*). Results pertaining to JW3606 (hep+) and JW3596 (hep−) are represented by blue and red boxes, respectively. Data were derived from three independent measurements for each condition tested. **c** Average size of TiO_2_NP−MV aggregates measured by DLS after 15 min co-incubation of TiO_2_NPs (50 mg/L) with MVs obtained from JW3606 (hep+) (blue boxes) and JW3596 (hep−) (red boxes), for different MV/TiO_2_NP concentration ratios at fixed 10 mM KNO_3_ background electrolyte. MVs were produced by the mutants incubated for 20 h in 10 mM KNO_3_ (in the absence of TiO_2_NPs), and they were subsequently collected after 0.45 μm filtration of the suspensions. MV concentration in (**c**) was changed upon diluting the filtrate with 10 mM KNO_3_ electrolyte (filtrate/KNO_3_ volume ratio). The gray box corresponds to the condition without MVs (KNO_3_ and TiO_2_NPs are only present in the solution). **d** Normalized amounts of JW3606 cells (hep+; TiO_2_NPs-sensitive strain) marked with propidium iodide (to target changes in membrane permeability) after 20 h co-incubation with TiO_2_NPs (50 mg/L) and MVs previously obtained from JW3606 (hep+) (blue boxes) and JW3596 (hep−) (red boxes) at different TiO_2_NP/MV concentration ratios, and fixed 10 mM KNO_3_ electrolyte (MVs were collected as in panel (**c**)). The gray box corresponds to the condition without added MVs (bacteria, KNO_3_ and TiO_2_NPs are present in the solution). In (**b**, **c**, **d**), data are reported in the form of box plots.

The presence of P2 particles is identified over the whole range of tested conditions (Fig. 3a), even in the absence of TiO_2_NPs. The intensity of the P2 peak increases with increasing TiO_2_NP concentrations from 0.5 to 50 mg/L. Given the positive mobility of TiO_2_NPs (Supplementary Fig. 1a, b), P2 necessarily refers to particles other than TiO_2_NPs, as confirmed by electrokinetic measurements on 0.22 μm-filtered cell−TiO_2_NP suspensions that are free of bacteria and TiO_2_NP aggregates (Supplementary Fig. 2a). Imaging of the filtrates by AFM further reveals that P2 particles are closed spheroids, that are polydisperse in size, with a diameter ranging from ca. 30–40 to 200 nm (Fig. 4a(i),(ii)), in accordance with refined measurements by dynamic light scattering (DLS) (Fig. 4a(iii)). These properties typically correspond to those of membrane vesicles (MVs for short) secreted by Gram-negative bacteria through the budding-out of their outer membrane^40^. Fluorescent labeling confirms the nature of the P2 particles (Fig. 5a(i),(ii)) and the significant increase of their produced amount with increasing TiO_2_NP concentration (Fig. 5b). To the best of our knowledge, these results establish for the first time the involvement of MVs in cell responses to TiO_2_NP stressors. Remarkably, DLS data (Fig. 4a(iii)) indicate that the mean diameter of secreted MVs increases from ca. 70 to 95 nm in the 1–10 mg/L TiO_2_NP concentration range before levelling off at higher concentrations (recalling that the 10–20 mg/L concentration regime is that where a significant loss of cell viability is reached, Fig. 2a). Finally, an increase in the MV concentration in the exposome above a threshold value via a short-term (15 min) co-incubation procedure (see details in “Methods”) leads to a decrease in the size of TiO_2_NP aggregates (Fig. 5c). This result demonstrates the existence of (electrostatically favored, Fig. 3 and Supplementary Fig. 1) MV−TiO_2_NP interactions, as further detailed in the “Discussion” section. The presence of MVs in solution further leads to a reduction in the TiO_2_NP-induced membrane permeabilization (Fig. 5d), thereby supporting the role of MVs in mitigating TiO_2_NP toxicity.

Following the above methodology, P1- and P2-contributions to electropherograms of JW3596 (hep−)−TiO_2_NPs (Fig. 3b) are attributed to JW3596 (hep−) and MVs, respectively (Figs. 4b, 5a(iii), (iv), and Supplementary Fig. 2b). The electrophoretic fingerprint of JW3596 (hep−) shifts slightly to negative values with increasing TiO_2_NP concentrations from 0 to 2 mg/L and practically vanishes at concentrations >2 mg/L (Fig. 3b). This contrasts with the JW3606 (hep+)−TiO_2_NP system for which the threshold TiO_2_NP concentration that marks the switch from a bacteria- to an MV-dominated *µ* distribution is ca. 20 mg/L (Fig. 3a). This difference is possibly due to the larger propensity of JW3596 (hep−) to aggregate^48^, as the absence of protruding surface LPS (Fig. 1) may reduce the magnitude of the stabilizing steric forces that are operational between neighboring cells. Like for JW3606 (hep+), MV production by JW3596 (hep−) in the absence of TiO_2_NPs is revealed by electrokinetics and AFM (Figs. 3b and  4b(i),(ii)), in accordance with their known vesiculation phenotypes^49^. Similarly to JW3606 (hep+), the mean diameter of MVs generated by JW3596 (hep−) increases with TiO_2_NP concentration with a ca. 20 nm increase over the whole range of tested conditions (Fig. 4b(iii)). Most importantly, MV production by JW3596 (hep−), unlike that by JW3606 (hep+), remains independent of TiO_2_NP concentration (Fig. 5b). Additionally, at sufficiently low TiO_2_NP concentrations (≤5 mg/L) and in the absence of TiO_2_NPs in solution, vesiculation by JW3596 (hep−) is much more important than that of JW3606 (hep+) (Fig. 5b). This latter finding correlates positively with the respective magnitudes of membrane permeability/depolarization and lipid peroxidation detailed for the two mutants in Fig. 2 at low TiO_2_NP doses. The larger amount of MVs secreted by JW3596 (hep−) further correlates with a more efficient reduction in the size of the TiO_2_NP aggregates (Fig. 5c) and a better membrane protection against TiO_2_NPs (Fig. 5d) in comparison with JW3606 (hep+).

### Nanomechanical properties of the *ΔrfaG* mutant envelope vary non-monotonously with TiO_2_NP concentration

**Fig. 6:**
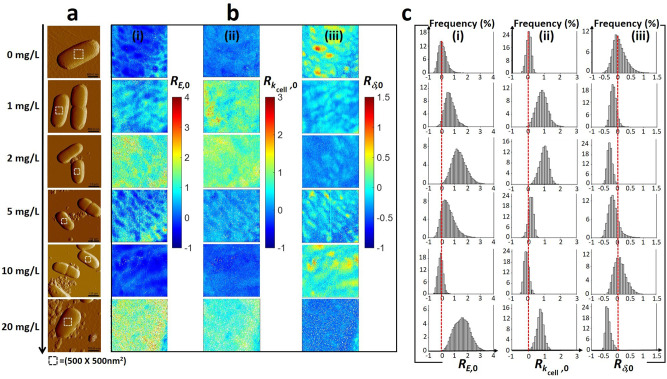
Multiparametric AFM evidences TiO_2_NP-mediated changes in cell surface elasticity and Turgor pressure for JW3606 (hep+) with increasing TiO_2_NP concentration. **a** Illustrative AFM deflection images of JW3606 (hep+) after 20 h co-incubation with TiO_2_NPs of various concentrations (indicated). The white boxes define the 500 × 500 nm^2^ cell surface areas where 65,536 force curve measurements were carried out. **b** Corresponding spatial maps of **(i)** Young modulus *E*, **(ii)** cell stiffness *k*_cell_, and **(iii)** indentation *δ* expressed in terms of ${R}_{E,0}$, ${R}_{{k}_{{\rm{cell}}},0}$and ${R}_{\delta ,0}$, respectively (see text for details). **c** Histogram-distributions of ${R}_{E,0}$, ${R}_{{k}_{{\rm{cell}}},0}$, and ${R}_{\delta ,0}$ corresponding to the maps given in (**b**). Red dotted lines in (**c**) are only guides to the eye.

**Fig. 7:**
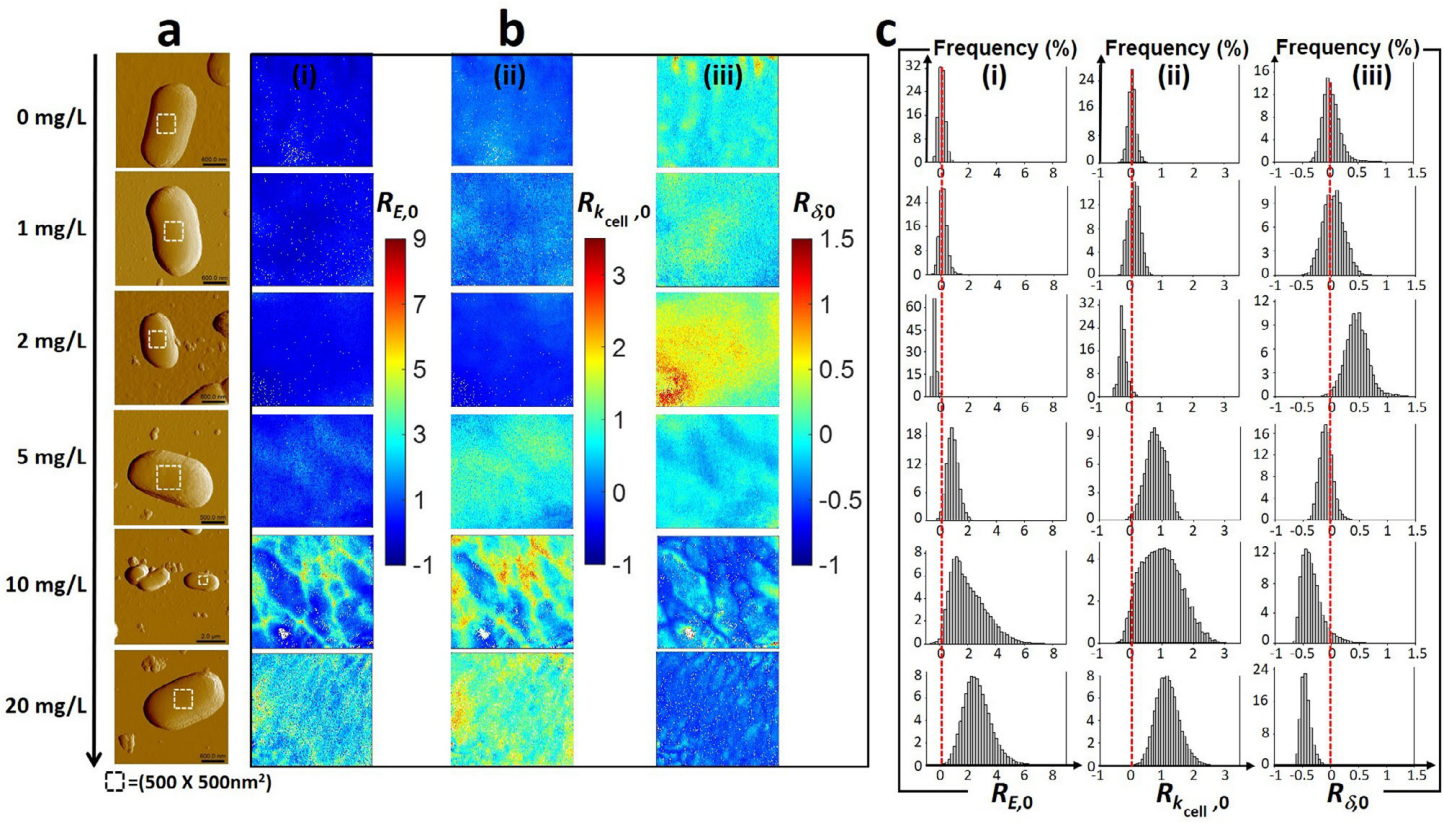
Multiparametric AFM evidences TiO_2_NP-mediated changes in cell surface elasticity and Turgor pressure for JW3596 (hep−) at larger TiO_2_NP concentrations than for JW3606 (hep+). **a** Illustrative AFM deflection images of JW3596 (hep−) after 20 h co-incubation with TiO_2_NPs of various concentrations (indicated). The white boxes define the 500 × 500 nm^2^ cell surface areas where 65,536 force curve measurements were carried out. **b** Corresponding spatial maps of **(i)** Young modulus *E*, **(ii)** cell stiffness *k*_cell_, and **(iii)** indentation *δ* expressed in terms of ${R}_{E,0}$, ${R}_{{k}_{{\rm{cell}}},0}$ and ${R}_{\delta ,0}$, respectively (see text for details). **c** Histogram distributions of ${R}_{E,0}$, ${R}_{{k}_{{\rm{cell}}},0}$ and ${R}_{\delta ,0}$ corresponding to the maps given in (**b**). Red dotted lines in (**c**) are only guides to the eye.

**Fig. 8:**
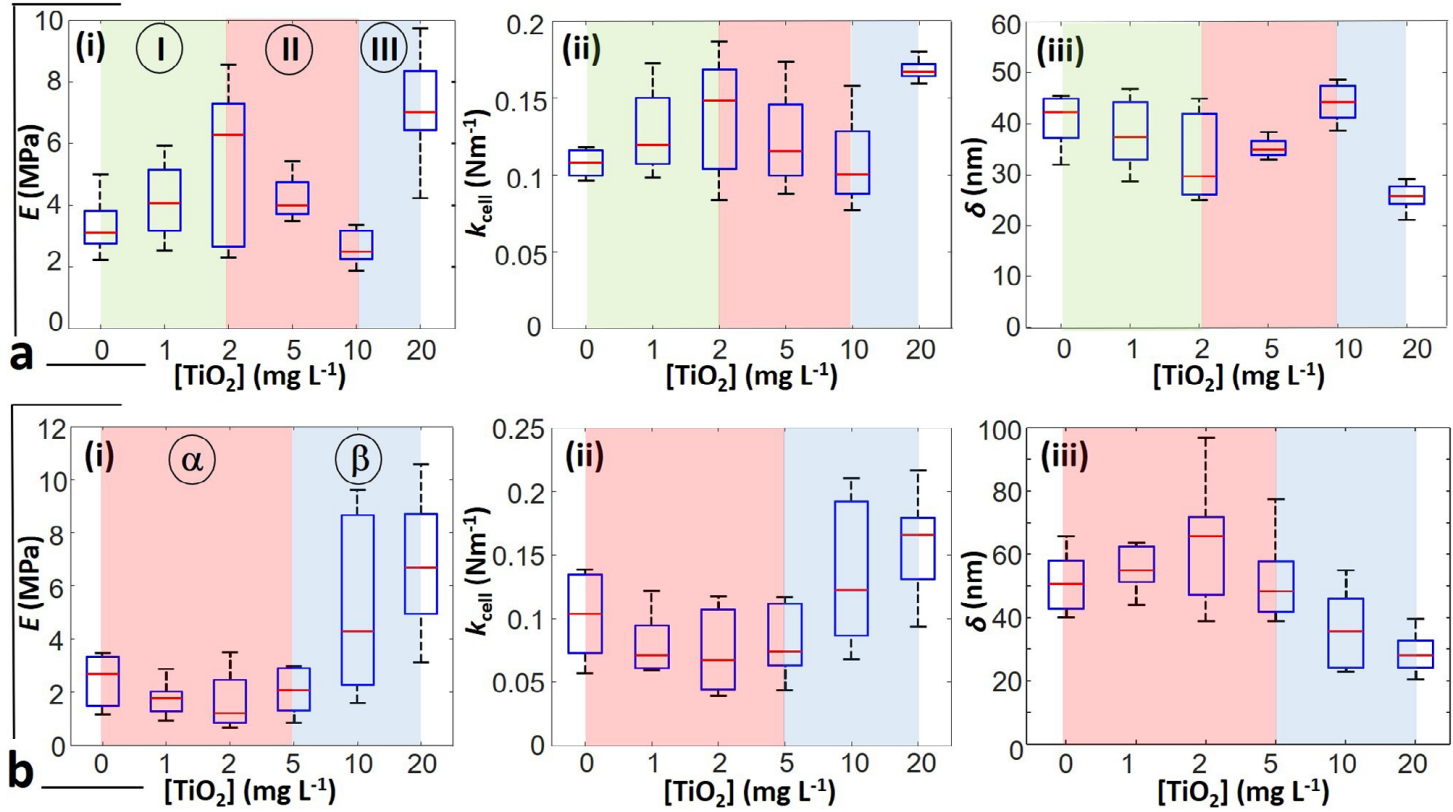
Nanomechanical properties of JW3606 (hep+), unlike those of JW3596 (hep−), vary non-monotonously with increasing TiO_2_NP concentration. Dependence of **(i)** Young modulus *E*, **(ii)** cell stiffness *k*_cell_, and **(iii)** indentation *δ* as a function TiO_2_NP concentration for **a** JW3606 (hep+) and **b** JW3596 (hep−) after 20 h exposure to TiO_2_NPs. Data correspond to distributions of the median values of *E*, *k*_cell_, and *δ* represented in the form of box plots and derived for each condition from measurements on *n* = 8 cells issued from different colonies or different cell cultures. Regimes I, II, III, α and β refer to distinct modes of action of TiO_2_NPs on JW3606 (hep+) and JW3596 (hep−), see details in the text. The red lines are the median of the plotted distributions. Statistical significance testing and *p*-values are provided in the Supplementary Information.

Spatial-distributions of the cell Young modulus (*E* in Pa) and cell stiffness (*k*_cell_ in Nm^−1^), indicative of the cell Turgor pressure^46,50^, were evaluated for (un)exposed JW3606 (hep+) and JW3596 (hep−) (Figs. 6a, b(i),(ii) and 7a, b(i),(ii), respectively) from theoretical analysis^50^ of 65,536 approach force curves collected by atomic force spectroscopy operated in PeakForce Tapping mode on 500 × 500 nm^2^ single-cell surface areas (see details in “Methods”). Below, we further introduce *δ* defined by the value of the indentation (in nm) which marks the transition between the non-linear elastic deformation of the cell envelope and the linear compliance domain in the force versus indentation curve measured at a given location (pixel) of the cell surface^50^. The spatial distribution of *δ* over the scanned cell surface area was obtained according to the theoretical procedure detailed elsewhere^50^, and it is reported in Figs. 6b(iii) and 7b(iii) for JW3606 (hep+) and JW3596 (hep−) exposed to different TiO_2_NP concentrations, respectively. The distributions of *E*, *k*_cell_, and *δ* values over the probed cell surface area are further provided in Figs. 6c−7c. For the sake of comparison, *E*, *k*_cell_, and *δ* derived as a function of TiO_2_NP concentration were converted into the normalized quantities *R*_*E*,0_, ${R}_{{k}_{{\rm{cell}}},0}$ and *R*_*δ*,0_ defined by ${R}_{X,0}=(X-{X}_{0})/{X}_{0}$ with *X* ≡ *E*, *k*_cell_, or *δ* and *X*_0_ the reference median value of the *X*-distribution measured at 0 mg/L TiO_2_NPs. Figure 8 finally collects the median values of the *E-*, *k*_cell_-, and *δ* -distributions derived as a function of TiO_2_NP concentration from measurements on eight cells from distinct grown colonies.

Results for JW3606 (hep+) evidence a synchronic and non-monotonous dependence of *R*_*E*,0_ and ${R}_{{k}_{{\rm{cell}}},0}$ on TiO_2_NP concentration (Fig. 8a(i),(ii)) with a *R*_*E*,0_-distribution width indicative of a spatial heterogeneity that is largest in the 1−2 mg/L range and at 20 mg/L (Fig. 6b, c). In detail, *R*_*E*,0_ and ${R}_{{k}_{{\rm{cell}}},0}$ increase from 0 to 2 mg/L (regime I), decrease with a further increase in the TiO_2_NP concentration up to 10 mg/L (regime II), and increase again from 10 to 20 mg/L (regime III).

The increase in *R*_*E*,0_ and ${R}_{{k}_{{\rm{cell}}},0}$ in regime I mirrors a stiffening of the cell envelope and a concomitant increase of the cell Turgor pressure. These trends compound a significant decrease of ${R}_{\delta ,0}$ (Fig. 6c) with a median *δ* value decreasing from 42 to 30 nm (Fig. 8a(iii)). The findings are consistent with a TiO_2_NP-mediated removal of the softer outer cell surface components, which leads to a reduction of the indentation range where non-linear deformation of the overall cell envelope is operational. The components removed by TiO_2_NP action in regime I likely include LPS, which is qualitatively supported by: (i) recent nanomechanics analysis of WT, JW3601, and JW3606 (Fig. 1) unexposed to NPs^43^, showing that the reduction in LPS length along this mutant gradient leads to increase in cell elasticity and stiffness, and (ii) TiO_2_NP-induced cell surface abrasion identified from JW3606 (hep+) electrokinetics (Fig. 3a).

In regime II, the decrease in *R*_*E*,0_ and ${R}_{{k}_{{\rm{cell}}},0}$, associated with an increase in ${R}_{\delta ,0}$, reflects a TiO_2_NP-mediated softening of the cell envelope and a decrease in Turgor pressure. The increase in ${R}_{\delta ,0}$ corresponds to an increase of the *δ* median from 30 to 45 nm. These different observations underpin a larger indentation into a mechanically softer biosurface (as compared to the regime I), in accordance with the loss of membrane integrity and increase in membrane permeability independently evidenced by flow cytometry for TiO_2_NP concentrations ≥2 mg/L (Fig. 2c). They further corroborate nanomechanics observations of *E. coli* exposed to SiO_2_NPs^51^. The drastic changes in membrane structure suggested by AFM in regime II are all the more favored as removal of the cell surface components in regime I has significantly weakened/disorganized the outer cell membrane, thereby rendering it more prone to TiO_2_NP-induced damage following lipid peroxidation (Fig. 2d) and oxidative stress (Fig. 2e). These processes lead to the leakage of intracellular ions and cell envelope components, which facilitates the formation of cohesive TiO_2_NP aggregate in the cell’s vicinity, as suggested by AFM imaging of cells for TiO_2_NP concentrations ≥5 mg/L (Fig. 6a).

In regime III, the increase in *E* and *k*_cell_ as the TiO_2_NP concentration increases from 10 to 20 mg/L, together with the decrease in *R*_*δ*,0_ (decrease in *δ* from 45 to 27 nm) highlight that the AFM probe now significantly interacts with the membrane components that are more rigid and subject to reduced indentation (Fig. 8a). Accordingly, we suggest that the successive TiO_2_NP-mediated LPS removal and outer membrane disruption taking place in regimes I and II lead to a significant AFM sensing of the rigid peptidoglycan layer in regime III. The heterogeneity in the bacterial surface landscape, reflected by the width of the *R*_*δ*,0_-distributions at the single-cell level (Fig. 6c), basically decreases with ongoing cell surface scouring (regime I), then it increases upon severe action of TiO_2_NPs on the outer membrane (regime II) and finally decreases significantly when the rigid peptidoglycan layer is significantly exposed and the outer membrane has significantly disintegrated (regime III). Close inspection of Fig. 6b indicates that details of *R*_*E*,0_-based surface heterogeneity do not necessarily match those inferred from ${R}_{{k}_{{\rm{cell}}},0}$-maps. This observation stems from the fact that *E* mostly reflects elastic properties of the peripheral cell surface envelope, whereas *k*_cell_ integrates properties of the whole membrane barrier via its connection to intracellular Turgor pressure^43,46^. Last, the spatially resolved *R*_*δ*,0_ property unveils irregular cell surface patterns in the absence of TiO_2_NPs, possibly connected to the surface distribution of protruding LPS^43,52^.

Concerning JW3596 (hep−), the dependence of ${R}_{E,0}$, ${R}_{{k}_{{\rm{cell}}},0}$ and ${R}_{\delta ,0}$ on TiO_2_NP concentration and their corresponding distributions at the cell surface differ from those derived for JW3606 (hep+) (Figs. 7, 8). Namely, ${R}_{E,0}$, ${R}_{{k}_{{\rm{cell}}},0}$ (${R}_{\delta ,0}$) medians slightly decrease (increases, respectively) with increasing TiO_2_NP concentrations from 0 to 2 mg/L (so-called regime α), but overlap in the statistical distributions (Fig. 8b) prevents firm conclusions from being drawn. In contrast, for TiO_2_NP concentrations >5 mg/L (regime β) ${R}_{E,0}$ and ${R}_{{k}_{{\rm{cell}}},0}$ (${R}_{\delta ,0}$) notably increase (decreases, respectively). The range of TiO_2_NP concentrations corresponding to regime β matches consistently the one where we measured a dramatic decrease in CFU (Fig. 2a) and the most significant changes in membrane potential (Fig. 2b), membrane permeabilization (Fig. 2c), lipid peroxidation (Fig. 2d), and oxidative stress (Fig. 2e). Regime β (5–20 mg/L) corresponds to a stiffening of the cell envelope and to an increase in cell Turgor pressure. These signatures are qualitatively similar to those described for JW3606 (hep+) in regimes I and III marked by cell surface abrasion and by increased contribution of the peptidoglycan layer to cell nanomechanics, respectively. The former process is detected by electrokinetics despite parasiting JW3596 (hep−) aggregation (Fig. 3b), and the latter requires a prior outer membrane alteration that is poorly supported by the slight decrease in ${R}_{E,0}$ and ${R}_{{k}_{{\rm{cell}}},0}$ in regime α (as compared to that in regime II for JW3606 (hep+)). Accordingly, we hypothesize that TiO_2_NPs predominantly impact on JW3596 (hep−) in regime β via cell envelope scouring (with resulting decrease in ${R}_{\delta ,0}$, Fig. 8b(iii)), accompanied by significant TiO_2_NP-induced increases in membrane permeability, membrane depolarization, lipid peroxidation, and oxidative stress. The heterogeneity of the so-modified cell surface is clearly identified from the maps in Fig. 7b at 10–20 mg/L TiO_2_NPs. Overall, multiparametric AFM at the single-cell level supports the results from macroscopic fluorescence-based assays: in comparison to JW3606 (hep+), JW3596 (hep−) is defined by a remarkable resistance phenotype against TiO_2_NPs.

### Transcriptomic analysis of deep rough mutants shows cell response to dominant osmotic stress

After 20 h exposure of JW3606 (hep+) and JW3596 (hep−) to 0–20 mg/L TiO_2_NPs, the expression of selected genes involved in osmotic and oxidative stress tolerance was quantified by RT-qPCR (see “Methods”).

**Fig. 9:**
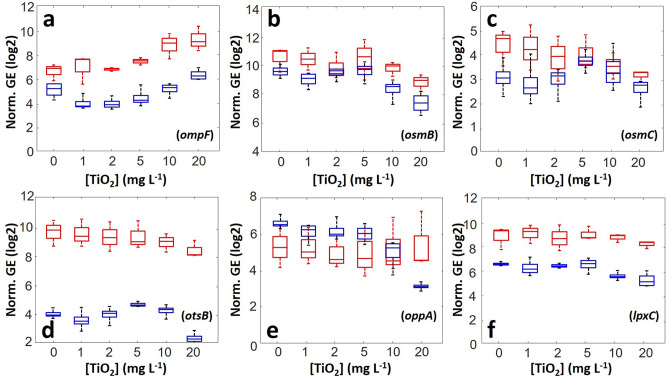
Transcriptomics evidences dominant osmotic stress generated by TiO_2_NPs. Expression levels of genes (**a:** *ompF*, **b:** *osmB*, **c:** *osmC*, **d:** *otsB*, **e:** *oppA*, **f:** *lpxC*) involved in the osmotic stress-response of JW3606 (hep+) (blue) and JW3596 (hep−) (red) as a function of TiO_2_NP concentration, and represented in the form of box plots (*n* = 4 for each condition tested). The normalized gene expression (norm. GE) was calculated from the ratio between the intensity of the targeted gene and the geometric mean intensity of reference genes. Statistical significance testing and *p*-values are provided in the Supplementary Information.

We first consider the osmotic stress-induced transcriptional response of JW3606 (hep+). Figure 9a evidences a dysregulation of the *ompF* gene that encodes OmpF protein which allows passive transport of small solutes across the membrane^53^. This gene is downregulated at 1–2 mg/L TiO_2_NPs, and its expression level increases with increasing TiO_2_NP concentrations from 2 to 20 mg/L, i.e., for doses where membrane permeability significantly increases (Fig. 2c). This non-monotonous *ompF* expression with varying TiO_2_NP concentration is strikingly reminiscent of that observed in regimes I−II for the Turgor pressure (Fig. 8a (ii)). In particular, reduction in *ompF* gene expression compounds the increase of the Turgor pressure in the 0–2 mg/L range, which is in accordance with the findings by Graeme-Cook^54^, who reported that *ompF* expression is switched off by Turgor stress. The *osmB* gene encoding an osmotic stress-inducible lipoprotein^53^ is further severely downregulated as the TiO_2_NP concentration increases from 5 to 20 mg/L, and so is the expression of *osmC*, another osmotic stress-induced gene (Fig. 9b, c)^53^. This marked downregulation is also observed for the *otsB* gene (Fig. 9d), which encodes a phosphatase involved in trehalose production to resist against osmotic stress^53^, for the *oppA* gene (Fig. 9e), which encodes a periplasmic binding protein of an ABC transporter that mediates high-affinity uptake of oligopeptides^53^, and to a lesser extent for the *lpxC* gene (Fig. 9f), which is known to play a regulatory role in lipid A biosynthesis^53^. The expressions of several genes encoding scavenger enzymes that protect cells against oxidative stress are further provided in Supplementary Fig. 3 for JW3606 (hep+). Among all tested genes, *sodB* and *ahpC* are those that are most significantly upregulated with increasing TiO_2_NP concentrations from 2–5 to 20 mg/L. They encode a superoxide dismutase and an alkyl hydroperoxide reductase, respectively, which are known to participate in the antioxidant defense mechanism against O_2_^•^- and H_2_O_2_-induced oxidative stress^53^ as detected by flow cytometry for TiO_2_NP concentrations ≥5 mg/L (Fig. 2e). Overall, the action of TiO_2_NPs at sufficiently low TiO_2_NP doses (<2–5 mg/L) mainly results in osmotic stress that couples to oxidative stress at higher concentrations. This finding is in line with literature^55–58^ suggesting that osmotic stress can leads to oxidative cell damage via disturbance of membrane components of the respiratory chain^58^.

A similar conclusion is obtained for JW3596 (hep−) cells (Fig. 9 and Supplementary Fig. 3) albeit with a few remarkable differences. Namely, *ompF* expression in JW3596 (hep−) remains stable up to a TiO_2_NP concentration of 2–5 mg/L and, similarly to Turgor pressure (Fig. 8b (ii)), it increases significantly at higher TiO_2_NP concentrations. Whereas the downregulation of *osmB* and *osmC* at high TiO_2_NP concentrations is a feature shared by JW3606 (hep+) and JW3596 (hep−), for this latter mutant the expression levels of *otsB*, *oppA,* and *lpxC* genes remain practically constant over the whole range of tested concentrations. These results imply that TiO_2_NPs impact the transcriptional response of JW3596 (hep−) to osmotic stress to a lesser extent than they do for JW3606 (hep+), in comparison to their respective controls (absence of TiO_2_NPs). Still, the overall magnitude of the osmotic stress, in the absence or presence of TiO_2_NPs, remains larger for JW3596 (hep−) (Fig. 9) than for JW3606 (hep+) as judged by the corresponding gene expressions levels. This finding correlates with the larger vesiculation capacity of JW3596 (hep−) either in the absence of nanoparticles or at sufficiently low TiO_2_NP concentrations (Fig. 5b), with their larger membrane permeability (Fig. 2c) and depolarization (Fig. 2b), and lipid peroxidation (Fig. 2d) in the 0–2 mg/L concentration range. Also, the TiO_2_NP-independent expressions of *katG* and *sodB*^53^ remain much lower than those for JW3606 (hep+) and only the transcription of *ahpC* is found to increase significantly at TiO_2_NP concentrations >5 mg/L (but with lower basal level compared to JW3606 (hep+), Supplementary Fig. 3e), in line with the oxidative stress detected under such concentration conditions (Fig. 2e).

## Discussion

**Fig. 10:**
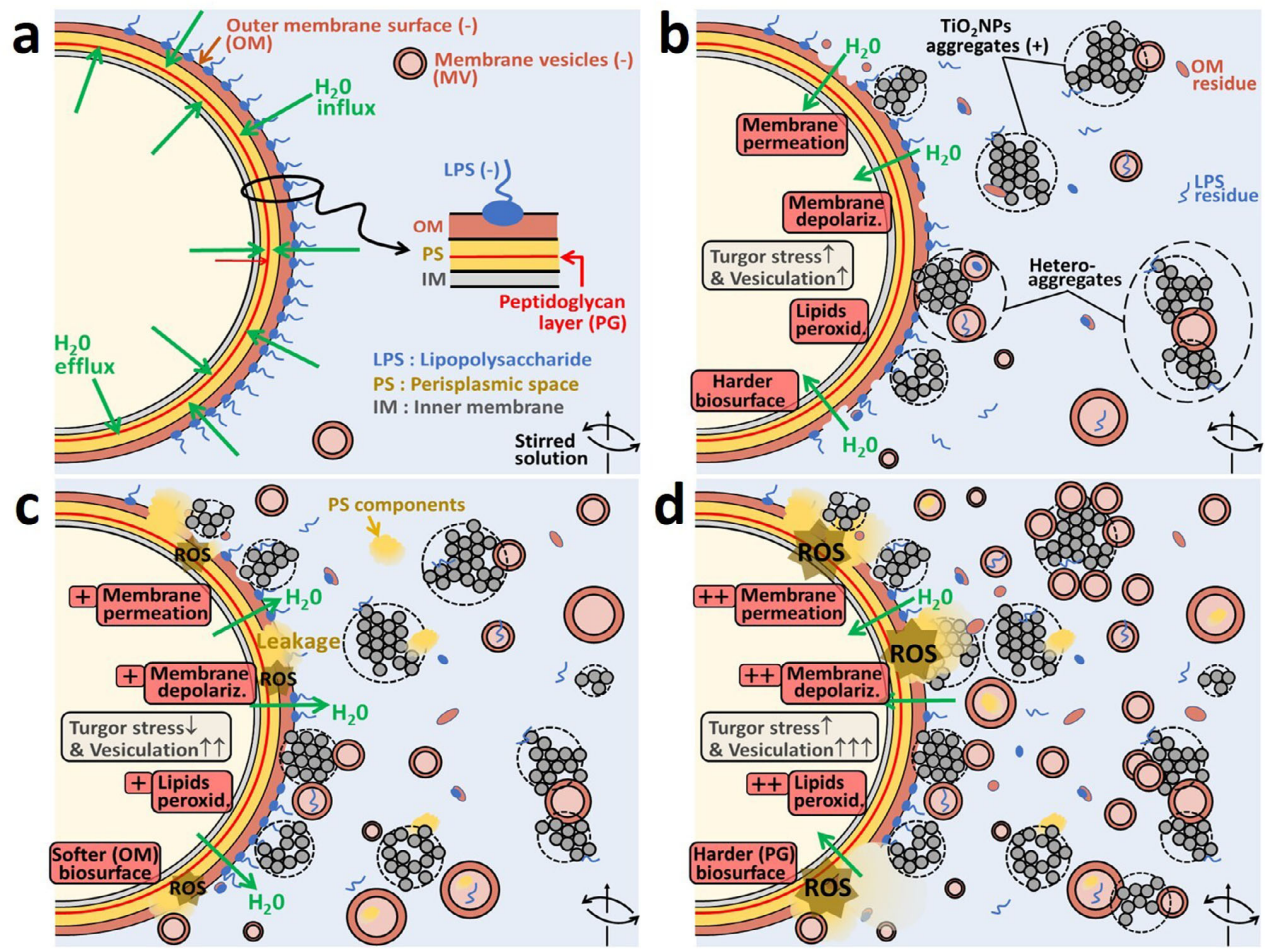
Schematic representation of TiO_2_NP toxicity mechanisms towards the most TiO_2_NP-sensitive JW3606 (hep+) mutant with un-truncated inner core LPS. Schematic overview (not to scale) of the modes of action of TiO_2_NPs on JW3606 (hep+). **a** Cells unexposed to TiO_2_NPs, and osmoregulation (water flux balance in the periplasm). **b** Regime I. **c** Regime II. **d** Regime III. Nomenclature: OM outer membrane, IM inner membrane, PS periplamic space, PG peptidoglycan layer, MV membrane vesicle, ROS reactive oxygen species. Green arrows indicate the water flux direction. (−) and (+) denote the sign of the charge carried by the OM surface, MVs and TiO_2_NPs. From (**a**) to (**d**), the TiO_2_NP concentration in the exposome gradually increases from 0 to 50 mg/L.

By a combination of cell viability, fluorescence, electrokinetic, nanomechanical, and transcriptomic analyses, we provide in Fig. 10 a schematic representation of the mechanisms that govern TiO_2_NP toxicity towards the most sensitive JW3606 (hep+) mutant, starting from the situation of Fig. 10a with cells featuring reduced vesiculation in the absence of TiO_2_NPs (Fig. 5b). For concentrations between 0 and 2 mg/L (regime I, Fig. 10b), TiO_2_NPs contribute to cell surface abrasion via removal of envelope components including LPS, and to a gradual exposure of the moderately altered outer membrane surface (Figs. 3a, 6, and 8a). Regime I is where cell osmoregulation that takes place in the absence of TiO_2_NPs (Fig. 10a) is inactivated by a growing destabilization of the outer membrane and the onset of membrane permeability increase (Fig. 2b, c). As a result, cell Turgor pressure increases (Figs. 6 and 8a(ii)) as a consequence of water entry under the selected hypotonic conditions, and the cell Young modulus increases (Fig. 8a(i)) due to the removal of the softest peripheral membrane components. The evidenced Turgor stress is further consistent with the downregulation of *ompF*, which prevents the additional entry of small hydrophilic solutes (Fig. 9a).

In regime I, MV production gently sets in with increasing TiO_2_NP concentration (Fig. 5b), and membrane integrity is not yet critically compromised (Fig. 2). Secreted vesicles probably mediate membrane stress relief via their evacuation of potentially harmful products, such as proteins or LPS, that accumulate in the periplasmic space with or without modification of the outer membrane-peptidoglycan linking lipoprotein levels^59^. Whereas the contribution of MVs to cell defense against e.g., antibiotics is well established^60^, their existence and roles have never been documented in the context of metallic oxide NP toxicity. In regime I, hyperproduction of MVs is not required to expel the moderate amount of residues that accumulate following LPS removal and associated alteration of the outer membrane.

With further increase in the TiO_2_NP concentration (regime II, 2–10 mg/L, Fig. 10c), the mechanical action of TiO_2_NPs on the cell surface intensifies and leads to significant loss of membrane integrity, increase in membrane permeability, and to marked membrane depolarization, oxidative stress and membrane lipid peroxidation (Fig. 2b−e). In turn, the underlying cell surface damage generates a pronounced cell envelop softening which is materialized by a decrease in cell surface elasticity and an increase of the threshold indentation that separates the non-linear deformation and compliance regimes (Figs. 6 and 8a(i),(iii)) in the AFM force−indentation curves. Also, a decrease in cell Turgor pressure (Figs. 6 and 8a(ii)) arises due to an increase in membrane permeability (Fig. 2c) and water efflux. The subsequent release of intracellular material favors NP aggregation (Fig. 6a). Cells then attempt to cope with the enhanced production of endogenic waste molecules in the periplasm via (i) an increase in the produced amount of MVs which act as shuttles to export over-accumulated moieties (Fig. 5b) that probably include lipid peroxidation products and LPS lipid A now loosely embedded in the disrupted outer membrane (Fig. 2d), and (ii) an increased expression of *ompF* (Fig. 9) that promotes passive uptake of solutes to counteract the significant leakage of intracellular ions. In relation to (i), MV size somewhat increases (Fig. 4a(iii)), which is consistent with the increased waste content to be released towards the extracellular medium. MVs further contribute to mitigate adverse TiO_2_NP effects (Fig. 5d) not only via expulsion of wastes generated by the deleterious action of TiO_2_NPs (direct effect), but also via their modification of the colloidal stability of TiO_2_NPs over time (depending on the concentration of secreted MVs, Fig. 5c) by electrostatically favored interactions between the MVs (negatively charged, Fig. 3 and S2) and TiO_2_NPs (positively charged, Supplementary Fig. 1a, b). This results in the formation of large TiO_2_NP−MV heteroaggregates and sedimentation thereof in the long term (indirect MV-mediated defense). Regime II is where significant loss of viability (Fig. 2a) is reached despite these established defense strategies.

At higher TiO_2_NP concentrations (regime III, >10 mg/L, Fig. 10d), deleterious effects of TiO_2_NPs in regime I and II have resulted in major outer membrane disruption, thereby exposing the thin rigid peptidoglycan layer in the periplasm to the outer aqueous environment, in agreement with nanomechanical measurements (Figs. 6 and 8a). In addition, all features delineated in regime II and derived from fluorescence-based assays (Fig. 2) are magnified in regime III, including the *ompF* over-expression (Fig. 9), consistent with a dramatic loss of membrane integrity and hyperproduction of MVs (Fig. 5b). Regime III is also where *osmB*, *otsB*, *oppA,* and *lpxC* genes (Fig. 9) are downregulated, probably due to the associated energy costs required to maintain the corresponding transcription at a stage where the cell viability is minimal. Also, it is in this regime that significant oxidative stress comes into play (Fig. 2 and Supplementary Fig. 3), triggered by osmotic stress^55–58^.

Concerning JW3596 (hep−), its nanomechanical properties remain practically unmodified in regime α (0–5 mg/L) (Figs. 7, 8b), in line with the absence of significant dependence of osmotic stress-responsive gene expression on TiO_2_NP concentration (Fig. 9). This feature is further in accordance with the absence of TiO_2_NP-induced lipid peroxidation and oxidative stress, with the preservation of membrane integrity and the maintenance of the membrane potential (Fig. 2). The higher native membrane permeability (i.e., at 0 mg/L TiO_2_NPs) of JW3596 (hep−) following inner core LPS truncation, as compared to JW3606 (hep+) (Fig. 2c), and the associated fragilization of the membrane^43^ offer an efficient way to circumscribe Turgor pressure perturbations caused by TiO_2_NPs without the need to significantly modulate the transcription of osmotic stress-responsive genes that are expressed at similar or higher levels compared to JW3606 (hep+) (Fig. 9). In addition, the significant vesiculation of JW3596 (hep−) in the absence of TiO_2_NPs (Fig. 5b) confers upon this mutant a more efficient defense, in line with the larger threshold TiO_2_NP concentration (5 mg/L) that marks the onset of significant harmful effects (Fig. 2). It is indeed only at concentrations >5 mg/L (regime β) that cell nanomechanical properties follow the trends discussed in regime I for JW3606 (hep+) (Figs. 7, 8b) and it is not until concentrations become higher than 5–10 mg/L that oxidative stress, membrane integrity loss, increase in membrane permeability, and lipid peroxidation become significant (Fig. 2). Over- and under-expressions of *ompF* and *osmB/osmC*, respectively, are also measured at TiO_2_NP concentrations >5 mg/L, as for JW3606 (hep+). In contrast, *otsB*, *oppA,* and *lpxC* expression levels are maintained constant under all tested exposure conditions, which reflects a more favorable energy balance than that for JW3606 (hep+).

In summary, our multiscale approach shows that *rfaG* gene mutation (JW3606 (hep+)) results in a moderate vesiculation capacity and a preserved membrane permeability in the absence of TiO_2_NPs. In turn, this dramatically reduces the efficiency of vesiculation and of osmoregulation cell strategies to circumvent the dominant osmotic stress induced by TiO_2_NPs at low concentrations. Our results demonstrate that a mutant with *rfaC* gene mutation and LPS truncation (and thus a membrane that is apparently more severely altered) resists the adverse effects of TiO_2_NPs in a much more efficient way due to its native hypervesiculation capability and significant regulatory response to osmotic stress in the absence of TiO_2_NPs. These results may shift current practice for fighting against harmful bacteria or preserving the viability of beneficial bacteria that are facing exogeneous contaminants. Indeed, controlled stress may initiate cell acquisition of weapons, such as vesicles, and thereby increase the cell defense arsenal against toxic contaminants. On a methodological level, the study introduces multiparametric atomic force microscopy/spectroscopy as a valuable tool to diagnose spatially resolved NP effects on biosurface nanomechanics at the single-cell level. It further integrates electrophoretic cell fingerprints within the context of NP toxicity evaluation at a level that goes beyond the traditional zeta-potential concept that is unapplicable to unravel electrokinetic properties of soft (ion- and flow-permeable) bacterial surfaces^45–47^. Finally, it succeeds to connect multiscale proxis (from the gene, single-cell to population scale) underpinning the various action modes of NPs (depending on the concentration in the exposome) and the corresponding cells response as derived by transcriptomics, cytometry, electrokinetics, and atomic force spectroscopy. Heterogeneities in cell response to TiO_2_NP exposure are further revealed at the gene, single-cell, and population scales.

## Methods

### Bacterial culture and preparation

*E. coli* strains BW25113 (wild type WT) and the knock-out *rfa*-gene mutants^41^ were obtained from the Coli Genetic Stock Center, Yale University. The position of the mutations in the *rfa* operons and the structure of the LPS resulting from these mutations are reported in Fig. 1. Knock-out mutations were checked by PCR before freezing at −80 °C in 50% glycerol solution. For experiments, cell cultures were first streaked from frozen stock on Luria Bertani (LB) agar (LB broth containing 1.5 w/v % agar) and incubated at 37 °C. Then, 4 mL preculture of M9 medium broth (6 g/L Na_2_HPO_4_, 3 g/L KH_2_PO_4_, 1 g/L NH_4_Cl, 0.5 g/L NaCl, 1 mM MgSO_4_, 0.1 mM CaCl_2_, 0.2% Glucose, 10 µg/mL Thiamine, 20 µg/mL Proline, and 25 µg/mL uridine) was inoculated with an isolated colony and incubated overnight at 37 °C under stirring. The next day, 100 mL of M9 medium broth were seeded at 1:100 dilution with the precultured cells and further incubated at 37 °C, 150 rpm, until the growth exponential phase was reached (OD_600nm_∼0.5). Cells were subsequently harvested by centrifugation (5000 × *g*, 5 min), washed twice with 10 mM KNO_3_. OD_600nm_ of the obtained bacterial suspensions was finally adjusted to 0.5 final value in 10 mM KNO_3_. Except for WT, all bacterial cultures were supplemented with kanamycin (30 mg/L) as a selective pressure.

### Titanium dioxide nanoparticles

Nanopowder of Aeroxide® TiO_2_ P25 was purchased from Evonik Degussa GmbH (Frankfurt, Germany). TiO_2_ nanoparticles (TiO_2_NPs) display an 80:20 anatase:rutile composition and are defined by a pristine particle radius of 21 nm and a specific surface area of 50 ± 15 m^2^/g according to the manufacturer’s information. Suspensions of TiO_2_NPs were prepared by dispersing 100 mg nanopowder in 10 mL sterile ultrapure water (milli-Q water, 18.2 MΩ cm) and were subsequently probe-sonicated (Sonics Vibra-cell 750 W, Sonics & Materials, frequency 20 kHz, 3 mm micro tip, amplitude 40%) in the dark for 30 min at 4 °C to break apart large TiO_2_NP aggregates and for homogenization purpose^26^. The so-prepared stock suspension of TiO_2_NPs (10 g/L concentration) was stable against aggregation-sedimentation for a month, and was protected from light.

### Bacteria exposure to nanoparticles

TiO_2_NP dispersions were prepared with concentrations in the range between 0.1 and 50 mg/L in 20 mL aliquots of bacterial suspensions (OD_600nm_∼0.5) at 10 mM KNO_3_ (pH∼5.5–6). Bacteria−TiO_2_NP mixtures were subsequently kept at 20 °C in the dark under 150 rpm stirring conditions for 20 h. For the sake of comparison, a reference sample containing bacteria unexposed to TiO_2_NPs was subjected to the same conditions. After 20 h, samples were analyzed using the procedures detailed in the sections below. In addition, bacteria−TiO_2_NPs suspensions were filtered with the use of a sterile vacuum filter bottle system with 0.45 µm or 0.22 µm porosity (Corning, CA membrane) to remove bacteria and TiO_2_NP aggregates. These filtrates were analyzed for evaluation of MVs charge/size properties and AFM imaging, as described below. To better identify the roles played by these MVs in mitigating TiO_2_NP toxicity, co-incubation experiments combining bacteria, 50 mg/L TiO_2_NPs, and filtrates at different dilution ratios in 10 mM KNO_3_ were carried out (Fig. 5c, d).

### Colony-forming unit (CFU)

The viability of bacterial cells exposed to TiO_2_NPs was assessed by CFUs per milliliter using the drop-count method^26^. The bacteria−TiO_2_NP mixtures were diluted serially at 1:100 to 1:10^5^. For each dilution condition, nine drops (20 µL per drop) were transferred onto the LB agar medium and incubated at 37 °C for 24 h. The percentage of viable cells was determined by comparing the number of CFUs obtained with exposed and unexposed samples.

### Fluorescence measurements

Cells exposed and unexposed to TiO_2_NPs were diluted 1:50 in 10 mM KNO_3_ and labeled using different fluorescent dyes. DIBAC_4_(3) (13.5 µM, 15 min at RT; Sigma Aldrich, Germany) was used to investigate TiO_2_NP effects on membrane depolarization, propidium iodide (30 µM, 15 min at RT; Life Technologies, USA) for evaluation of membrane permeability, BODIPY (2.5 µM, 15 min at RT; Life Technologies, USA) for that of lipid peroxidation, H_2_DCFDA (2 µM, 15 min at RT; Sigma Aldrich, Germany) to address oxidative stress, and the membrane-selective dyes FM4-64 (5 µg/ml, 15 min at RT; Invitrogen, USA) and Syto9 Green Fluorescent Nucleic Acid Stain (5 µM, 15 min at RT; Life Technologies, USA) for cell numeration. Labeled samples were then analyzed by flow cytometry on a BD Accuri™ C6 and a BD Biosciences (BD Biosciences, New Jersey, USA) equipped with a laser emitting at 488 nm. Forward scatter (FSC), side scatter (SSC), and Syto9 signal on FL1 channel (530 nm) or FM4-64 signal on FL3 channel (LP 670 nm) were used to discriminate bacteria and nanoparticle aggregates from the background, and the trigger was set at 15,000 on FSC. For detection of MVs with the use of FM4-64 labeling, the trigger was set at 1000 on FL3 and 100 on FSC (Fig. 5a). DIBAC_4_(3), BODIPY, H_2_DCFDA, and Syto9 fluorescence were recorded on the FL1 channel and propidium iodide on the FL2 channel (585 nm). For each sample, at least 20,000 events in the gate corresponding to the bacteria were collected in SSC versus FSC dot plot. The Accuri^TM^ cytometer is equipped with peristaltic pumps that allow sample volume measurement and, therefore, accurate determination of cell concentration. Acquisition and further analysis were performed with BD Accuri^TM^ software (BD Biosciences). Each set of experiments was repeated at least three times to ensure data reproducibility. In addition, due to the detection limit of the flow cytometer (i.e., >200 nm), quantitative fluorescence measurements were performed using a plate‐reader fluorometer (SAFAS, Monaco) to determine the relative amount of MVs (Fig. 5b). These experiments were performed on filtrates (see “Bacteria exposition to nanoparticles” subsection above) using a DNA stain-binding assay because it was previously reported that MVs contain DNA^40^. For that purpose, after 20 h exposure, cell suspensions were 0.45 μm filtrated and treated with DNase I (10 U/mL, 20 min at 37 °C; Sigma Aldrich) to remove extracellular DNA associated with MVs, and subsequently stained with Syto9 (5 µM, 30 min at RT). After excitation at 485 nm, the emission at 502 nm was measured on three replicate samples. The amount of MVs in the filtrates, expressed in relative fluorescence units, was determined after subtracting the control (i.e., Syto9 probe alone in 10 mM KNO_3_).

### Electrokinetics and particle size measurements

After 20 h, samples containing bacteria exposed and unexposed to TiO_2_NPs were diluted at 1:10 in ultrapure water, leading to cells suspended in 1 mM KNO_3_ background electrolyte. For each TiO_2_NP concentration tested, the electrophoretic mobility distributions (electropherograms) of so-prepared bacteria−TiO_2_NP suspensions were measured at natural pH and room temperature using a Zetaphoremeter IV (CAD Instrumentations, Les Essarts le Roi, France). Electrophoretic mobility evaluation consisted of following the displacements of particles in a quartz Suprasil^®^ rectangular capillary upon application of a constant direct-current electric field (800 V/m) and particle tracking was monitored by the reflection of a laser beam at 90° angle with the use of a charge-coupled device camera. Trajectories were recorded in real-time and processed by CAD image analysis software to derive electrophoretic mobility distributions. For each tested condition, particle displacements generated by the applied electric field were collected from three replicates with aliquots prepared from a given bacteria−TiO_2_NP preparation, and independent sets (*n* = 3−14, Fig. 3) of three measurements were further performed for each condition starting from different cell cultures and cell colonies. Electrophoretic mobility distributions of the filtrates (see “Bacteria exposure to nanoparticles” subsection) were measured following the above procedure (Supplementary Fig. 2). Distributions of the hydrodynamic diameter of the particles dispersed in the filtrates were collected with a Zetasizer NanoZS equipment (Malvern Panalytical, He−Ne red laser, 633 nm) by DLS. In detail, particle diffusion coefficients were measured and converted into hydrodynamic size on the basis of the Stokes−Einstein equation. For each tested condition, three measurements were carried out in a row, and independent measurement sets (*n* = 2−6, Fig. 4) of such three measurements were also performed on samples prepared from different cell suspensions issued from distinct colonies. Distributions of hydrodynamic size and electrophoretic mobility pertaining to only the TiO_2_NPs in KNO_3_ electrolyte solution (pH∼5.5) were further measured in 10 mM KNO_3_ background electrolyte (Supplementary Fig. 1). While TiO_2_NP size measurements were performed following the protocol detailed above, their electrophoretic mobility was measured with Zetasizer NanoZS device by phase analysis light scattering.

### Atomic force microscopy (AFM) and force spectroscopy measurements

Bacteria were deposited onto a cleaned borosilicate glass slide previously covered by a polyethyleneimine layer (Sigma, Mw = 750,000 g/mol) as detailed elsewhere^43^. A few minutes after cell deposition, the glass slide was rinsed with 1 mM KNO_3_ solution to remove unbound bacteria, and the remaining bacteria on the surface were kept in a 1 mM KNO_3_ environment (5 ml drop) prior to AFM experiments. Nanomechanical AFM measurements were performed with a FastScan Dimension Icon and Nanoscope V controller (Bruker) operating in PeakForce Tapping mode at room temperature in 1 mM KNO_3_ electrolyte. Adopted AFM probes were NPG Silicon Nitride tips with 20–30 nm curvature radius and a nominal spring constant of 0.24 N/m (0.12–0.48 N/m range) as provided by the manufacturer. Prior to each measurement, a calibration was performed on the rigid substratum to determine the deflection sensitivity (nm/V) of the AFM probe and the cantilever spring constant by the thermal tune method^61^, with a resulting value of 0.40 ± 0.2 N/m. Force measurements were recorded during the approach and retraction of the AFM probe to the bacterial surface. The pixel-by-pixel force curves were recorded at the apex of the cell with 500 nm scan size (256 × 256 local force curve measurements at 1 Hz scan rate and 1 μm/s probe velocity). The setpoint adopted for all force measurements was 5 nN. As previously shown^46^, the liquid environment adopted for the AFM measurements (1 mM KNO_3_) allows a proper detection of changes in cell surface structure from modulations of the cell Young modulus (elasticity) and cell Turgor pressure (related to the cell stiffness) derived here by analysis of the approach force curves along the lines detailed elsewhere^50^. Briefly, nanomechanical cell properties (*E*, *k*_cell_) and indentation *δ,* which marks the transition between the non-linear part of the force-indentation curve and the linear compliance regime, were evaluated on the basis of the Sneddon model corrected for finite cell thickness and Hook’s law using a home-made MATLAB program able to handle rapidly the analysis of a large number of force curves (65,536 here per cell examined, with *n* = 8 probed cells issued from similar and different colonies, Fig. 8)^50^. All reported spatial maps are based on only the force curves that were successfully fitted with *R*^2^ > 0.95^50^, and curves that did not comply with this condition were systematically rejected (white dots in Figs. 6, 7). Cell elasticity and stiffness were derived for TiO_2_NP concentrations in the range 0–20 mg/L as measurements at higher concentrations were significantly impaired by AFM probe contamination by TiO_2_NPs. AFM imaging of bacteria and filtrates (see “Bacteria exposure to nanoparticles” subsection above) was also performed by PeakForce Tapping mode that best preserves the integrity of fragile biosurfaces upon probe scanning. The first-order estimation of the size distribution of MVs obtained by 0.45 µm-filtration of bacteria−TiO_2_NP suspensions and subsequently deposited (50 μl) on a cleaned borosilicate glass slide, was derived (after sample drying) from AFM images collected in air and analyzed with WSXM free software^62^. For that purpose, based on literature results^40^ a minimum cutoff MV diameter of 20 nm was selected and a minimal value was further imposed for the height of the particles to be included in the analysis. As the size evaluation of soft MV particles deposited on a rigid surface and imaged after drying in the air is necessarily approximate due to e.g., capillarity-driven particle deformation, DLS measurements were further conducted to refine MV size estimation in aqueous solution (Fig. 4).

### Transcriptomics

Bacteria−TiO_2_NP suspensions were centrifuged (7000 × *g*, 10 min) and pellets were stored at −80 °C. RNA extractions were performed using an UltraClean Microbial RNA isolation kit (MOBIO, CA, USA). After extraction, contaminating DNA was digested with DNase I (Sigma Aldrich), and total RNA was purified by phenol/chloroform extraction and ethanol precipitation. RNA quantity and purity were assessed by OD measurements (OD_260nm_ and ODs ratio 260nm/280 nm and 260 nm/230 nm) and RNA integrity was checked using Bioanalyseur 2100 (Agilent, CA, USA). The cDNA was synthesized in a final volume of 20 µl using 550 ng of RNA, 2.5 µM of random hexamer primers, and SuperScript^®^ IV reverse transcriptase according to the manufacturer’s instructions (Invitrogen). RT-qPCR was performed on 12 selected genes that encode enzymes involved in ROS scavenging and osmotic stress regulatory pathways. Genes and primers are listed in the Supplementary Information, Supplementary Table 1. Primers were designed using Primer3Plus^63^. The qPCR reaction was conducted with 2 µL of cDNA (30 ng/reaction) as a template, 150 or 250 nM primers and Fast SYBR^®^ green master mix (Applied Biosystem^®^, CA, USA) in a reaction mixture with a final volume of 20 µL. The cycling conditions were 20 s at 95 °C, followed by 40 cycles of 3 s at 95 °C and 30 s at 60 °C. Amplification efficiencies (between 90 and 110%) of all primers were verified and amplicon sizes were also verified on agarose gel. All PCR amplifications were performed in four biological replicates using the StepOnePlus RT-PCR system (Applied Biosystems^®^). Gene expression levels (Fig. 9−Supplementary Fig. 3) were analyzed using the relative quantification method (∆∆Ct)^64^. In order to select a suitable reference gene, the stability of five genes was tested on 12 cDNA produced from 12 cell cultures exposed to different concentrations of TiO_2_NPs and analyzed with Genorm^65^. *IhfB* and *idnt* were assigned as the most stable genes and these genes have already been used several times as references^13,66–68^. The ∆Ct and the pooled standard deviation were calculated by normalizing the gene of interest Ct value by the geometric mean of the two Ct from reference genes.

### Statistics and reproducibility

Data reported in Figs. 2, 8, 9, and Supplementary Fig. 3 were statistically analyzed with R software, version 4.0.3. Data were first tested using the Shapiro−Wilk test for normality and the Bartlett test for homogeneity of variances. Based on the outcome of these tests, we used either a parametric one-way ANOVA followed by Tukey post hoc test or a non-parametric Kruskal−Wallis ANOVA with Dunn post hoc test. Post hoc tests were only performed when the overall ANOVA or Kruskal−Wallis ANOVA revealed overall significance. Statistical results are provided in Supplementary Tables 2–5. Statistical testing of the data was performed by excluding the few outliers of the box plots. Note that the statistical analysis (i) should be cautiously considered for appreciation of the significance of cytometry and transcriptomic measurements carried out with *n* = 3−4 independent replicas, as commonly done in literature (Figs. 2, 9 and Supplementary Fig. 3) due to the dependence of the test outcome on *n*, and (ii) does not inform on the significance of the overall data dependence on TiO_2_NP concentration (including that of their distribution widths, which reflects multiscale heterogeneities in cell response to TiO_2_NP stressors). The numbers of replicates adopted in this work for the various experiments conform to what is classically reported in the literature.

### Reporting summary

Further information on research design is available in the Nature Research Reporting Summary linked to this article.

## Supplementary information

Supplementary Information

Description of Supplementary Files

Supplementary Data

Reporting Summary

## Data Availability

The authors declare that the data supporting the findings of this study are available within the paper and its supplementary information file. All source data underlying the graphs presented in Figs. 1–9 are made available as Supplementary Data with accompanying captions. All other data are available from the corresponding author on reasonable request.

## Code availability

All codes used for the analysis of the data within this paper are available upon request to the authors.
