# Supplementary material for: Osmotic stress and vesiculation as key mechanisms controlling bacterial sensitivity and resistance to TiO2 nanoparticles
Source: Commun Biol. 2021 Jun 3;4:678. doi: 10.1038/s42003-021-02213-y (PMC8175758; doi:10.1038/s42003-021-02213-y)
Supplement: Supplementary file 1 — Supplementary Information [file 42003_2021_2213_MOESM1_ESM.pdf]

SUPPLEMENTARY INFORMATION

Osmotic stress and vesiculation as key mechanisms controlling bacterial sensitivity and resistance to TiO_2 nanoparticles

Christophe Pagnout,[1] Angelina Razafitianamaharavo,[2,#] Bénédicte Sohm,[1,#] Céline Caillet,[2,#]

Audrey Beaussart,[2] Eva Delatour,[1] Isabelle Bihannic,[2] Marc Offroy,[2] Jérôme F.L. Duval[2,]*

[1] Université de Lorraine, CNRS, LIEC, F-57000 Metz, France.
[2] Université de Lorraine, CNRS, LIEC, F-54000 Nancy, France.

* Corresponding author: jerome.duval@univ-lorraine.fr

[#] Equally contributed to this work

This document contains 3 Supplementary Figures and 5 Supplementary Tables. 22 Supplementary data with accompanying captions are available in the form of excel files on *Nature* website.

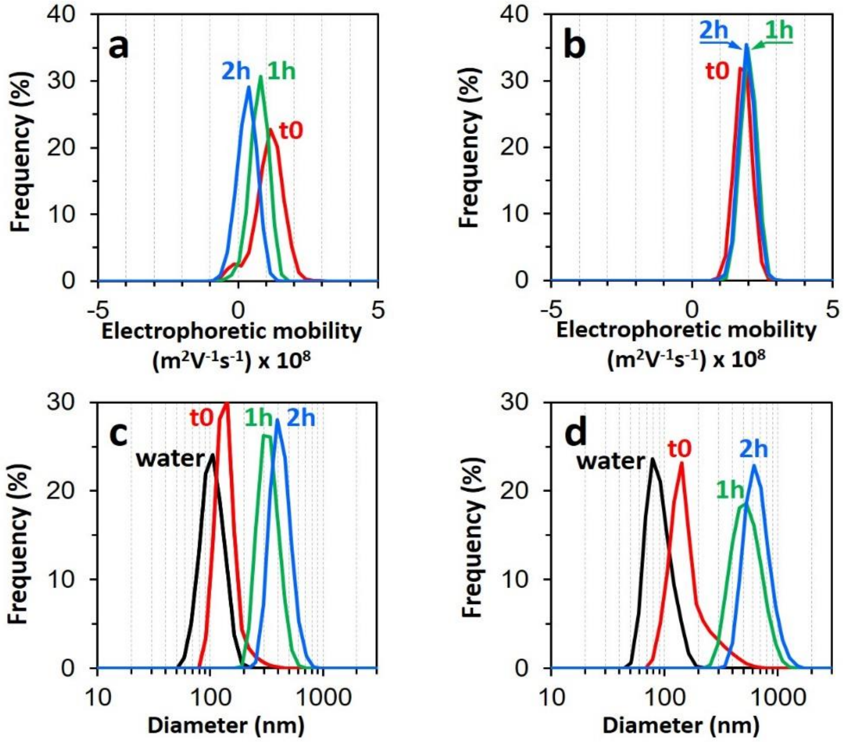

Supplementary Figure 1. a,b: Electrophoretic mobility and **c,d:** size distributions of TiO_2NPs in 10 mM KNO_3 electrolyte and in ultrapure water (specified) as a function of time (indicated) at **a,c:** 1 mg/L and **b,d:** 10 mg/L TiO_2NP concentration. 't0' refers to measurements immediately after homogenization of the TiO_2NP suspensions in 10 mM KNO_3 electrolyte, and subsequent measurements after 1 h and 2 h are indicated. Error bar for most frequent size and mobility values: 20%.

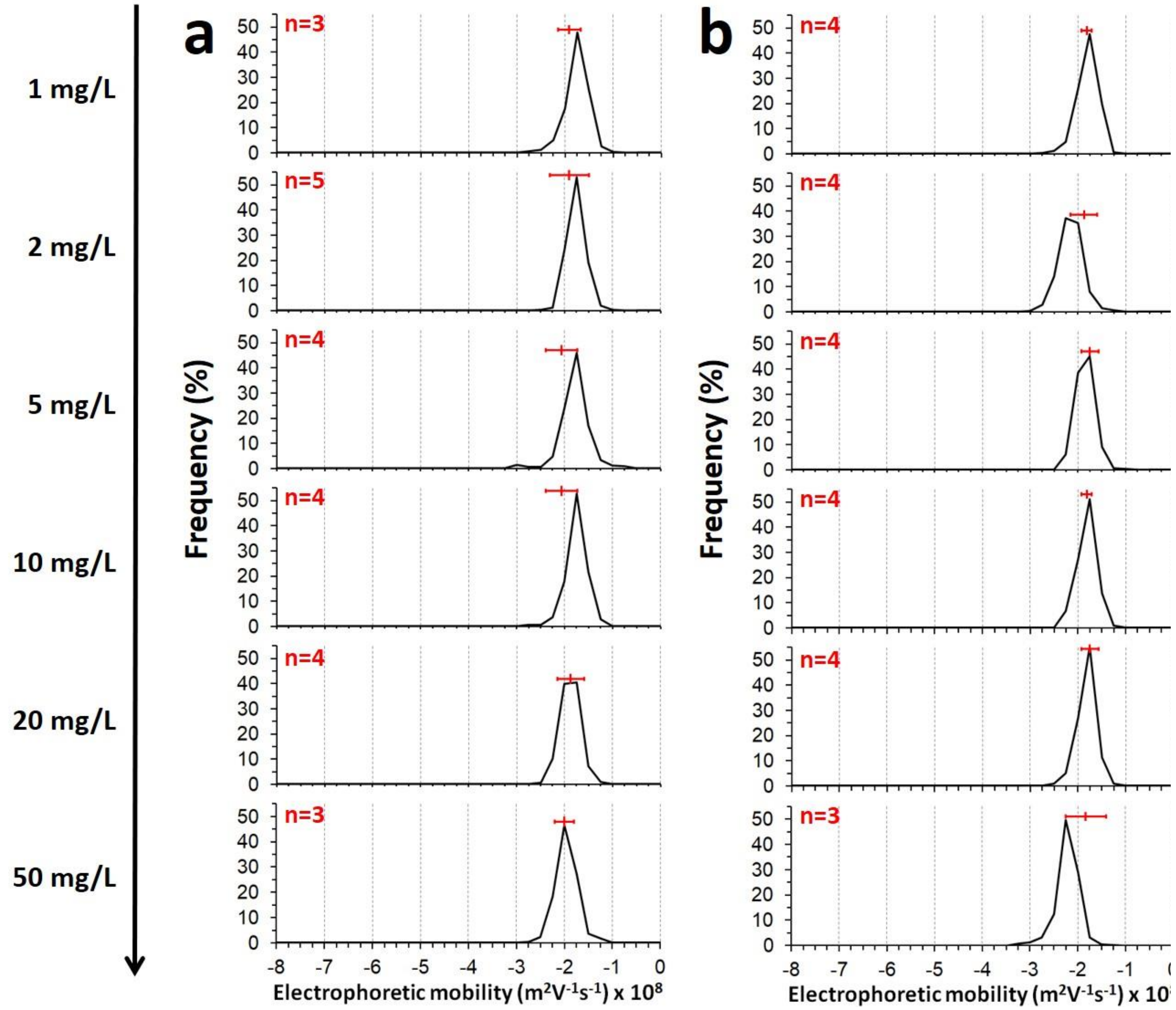

Supplementary Figure 2. a,b: Electrophoretic mobility distributions measured for filtrates obtained by 0.22 μm-filtering of suspensions (and 1:10 dilution in ultrapure water, see *Methods*) of **a:** JW3606 (hep+) and **b:** JW3596 (hep-) incubated in 10 mM KNO_3 electrolyte solution (pH 5.5) for 20 h in the presence of TiO_2NPs at various concentrations (indicated). Horizontal red bars correspond to position of the maxima (mean values ± standard deviations) derived from the measurement of *n* (indicated) electrophoretic mobility distributions for suspensions prepared from different cell cultures or colonies, with 3 replicates for each measurement. The reported illustrative distributions are averaged over 3 replicates.

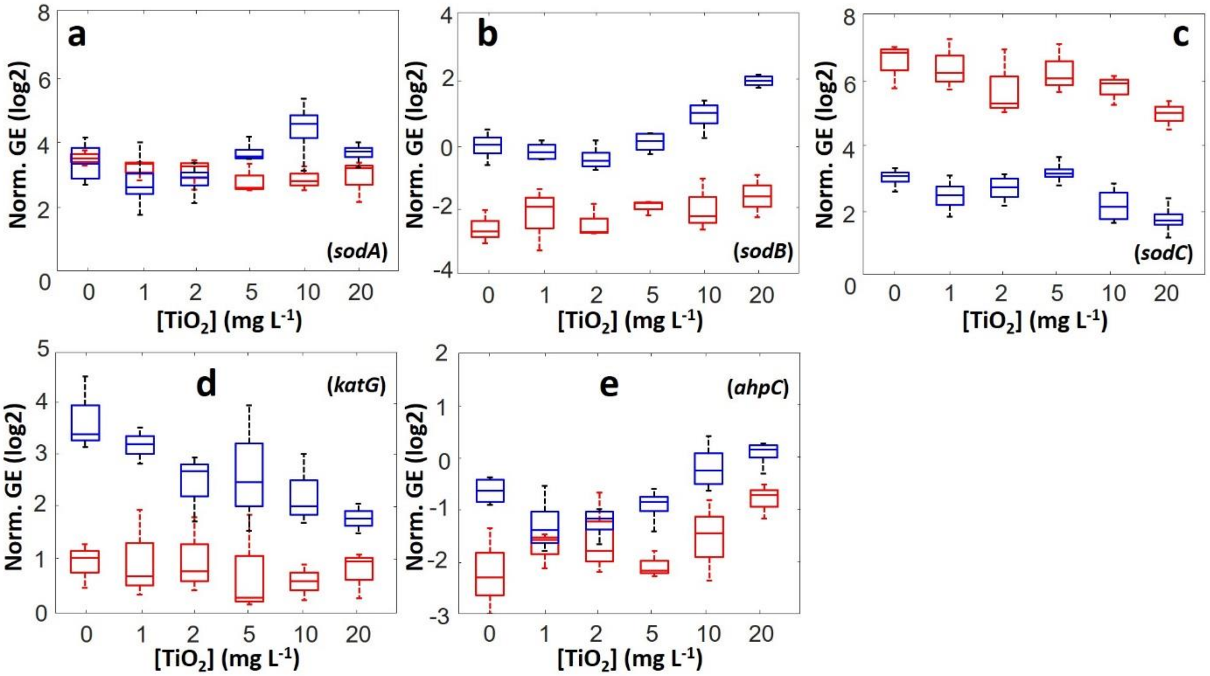

Supplementary Figure 3. Expression levels of genes (specified in each panel) involved in the oxidative stress-response of JW3606 (hep+) (blue) and JW3596 (hep-) (red) as a function of TiO_2NP concentration, and represented in the form of box plots (*n*=4 for each condition tested). The normalized gene expression (norm. GE) was calculated from the ratio between intensity of targeted gene and geometric mean intensity of reference genes (see *Methods*). Statistical significance testing and p-values are provided in the Supplementary Information .

Supplementary Table 1. Summary of genes primer sequences (5’-3’), amplicon size and primer final concentration used in RT-qPCR.

Gene name	Forward primer	Reverse primer	Amplicon size (pb)	Conc. used for qPCR (nM)
ahpC	TGCGTGAAGATGAAGGTCTG	TCGATTGCCTGGATGATACC	73	150
idnt	TCCCGCTTTAATGGTACTGG	ACGGCGTTAATGGCTAACAC	186	150
ihfB	CAAGACGGTTGAAGATGCAG	TTATCGCCAGTCTTCGGATT	150	150
katG	CTGGTGTGGTTGGTGTTGAG	CGTCAGTTGCTGTGCTTTGT	215	250
lpxC	GCCAAATCTGTGCGTGATAC	GAGGTGCTCTACGGTTGAAATC	80	250
ompF	AGGCTTTGGTATCGTTGGTG	TGCCAGGTAGATGTTGTTCG	136	250
oppA	ACACTGGCGAAAAACAAAC	ACCATTTCGCATCTTTACGC	226	250
osmB	TTGGCAATGTCTCTGAGTG	CACCACCTAATGTACCCAAC	130	150
osmC	AAGGCGAAAAAGGAACCAAC	CACCGCAACTTCACTCTTCA	197	250
otsB	ACAGTCATCGCTCAGTATCC	CACACTTTCCCTGCTGTAAC	160	150
sodA	GGAAATCCACCACACCAAAC	GATAGCCGCTTTCAGGTCAC	238	250
sodB	AAAAACAGCGATGGCAAACT	AGTGCTCCAGATAGCCAGGA	151	250
sodC	CAGTCAATTGGTAGCGTCAC	CTGGCCCTTCATGTTTAC	208	150

Supplementary Table 2. Statistical significance testing for data presented in **Figure 2**. ****p*<0.001; ***p*<0.01; **p*<0.05. The left column indicates concentrations of TiO_2NPs in mg/L. All means data are expressed in % (cf. Figure 2).

JW3606

CFU

	Means	0	1	2	5	10	20
0	99						
1	99	1.0000					
2	99	1.0000	1.0000				
5	94.67	0.9983	0.9983	0.9983			
10	33.73	**0.0001*****	**0.0001*****	**0.0001*****	**0.0001*****		
20	25.87	**0.0001*****	**0.0001*****	**0.0001*****	**0.0001*****	0.9627	
50	6.97	**0.0001*****	**0.0001*****	**0.0001*****	**0.0001*****	0.0817	0.3470

Dibac$_4$(3)

	Means	0	1	2	5	10	20
0	3.33						
1	3.2	1.0000					
2	17.87	0.1995	0.1922				
5	51.37	**0.0001*****	**0.0001*****	**0.0005*****			
10	64.87	**0.0001*****	**0.0001*****	**0.0001*****	0.2635		
20	89.27	**0.0001*****	**0.0001*****	**0.0001*****	**0.0001*****	**0.0091****	
50	99.67	**0.0001*****	**0.0001*****	**0.0001*****	**0.0001*****	**0.0004*****	0.5356

PI

	Means	0	1	2	5	10	20
0	7.03						
1	4.67	1.000					
2	13.57	0.9921	0.9632				
5	46.23	**0.0160***	**0.0102***	0.0540			
10	62.10	**0.0009*****	**0.0006*****	**0.0028****	0.6631		
20	72.00	**0.0002*****	**0.0001*****	**0.0005*****	0.1802	0.9403	
50	99.80	**0.0001*****	**0.0001*****	**0.0001*****	**0.0011****	0.0212	0.1283

BODIPY

	Means	0	1	2	5	10	20
0	4.93						
1	9.33	0.9987					
2	24.20	0.3870	0.6592				
5	66.67	**0.0001*****	**0.0003*****	**0.0049****			
10	68.60	**0.0001*****	**0.0002*****	**0.0033****	1.0000		
20	89.17	**0.0001*****	**0.0001*****	**0.0001*****	0.2345	0.3193	
50	95.57	**0.0001*****	**0.0001*****	**0.0001*****	0.0734	0.1061	0.9898

H_2DCFDA

	Means	0	1	2	5	10	20
0	1.4						
1	0.65	1.0000					
2	1.1	1.0000	1.0000				
5	9.53	0.8497	0.7927	0.8279			
10	36.0	**0.0006*****	**0.0004*****	**0.0005*****	**0.0145***		
20	34.4	**0.0009*****	**0.0007*****	**0.0009*****	**0.0236***	1.0000	
50	99.4	**0.0001*****	**0.0001*****	**0.0001*****	**0.0001*****	**0.0001*****	**0.0001*****

JW3596

CFU

	Means	0	1	2	5	10	20
0	99.33						
1	99.67	1.0000					
2	98.67	1.0000	1.0000				
5	97.33	0.9995	0.9988	1.0000			
10	70.23	**0.0006*****	**0.0005*****	**0.0007*****	**0.0011****		
20	42.4	**0.0001*****	**0.0001*****	**0.0001*****	**0.0001*****	**0.0009*****	
50	13.5	**0.0001*****	**0.0001*****	**0.0001*****	**0.0001*****	**0.0001*****	**0.0006*****

Dibac$_4$(3)

	Means	0	1	2	5	10	20
0	22.07						
1	18.2	0.9997					
2	18.03	0.9996	1.0000				
5	15.73	0.9949	1.0000	1.0000			
10	9.9	0.8867	0.9797	0.9816	0.9968		
20	24.17	1.0000	0.9963	0.9957	0.9780	0.7955	
50	74.7	**0.0022****	**0.0011****	**0.0011****	**0.0007*****	**0.0003*****	**0.0031****

PI

	Means	0	1	2	5	10	20
0	12						
1	11.83	1.0000					
2	12.35	1.0000	1.0000				
5	7.08	0.9955	0.9963	0.9935			
10	12.18	1.0000	1.0000	1.0000	0.9946		
20	26.23	0.5743	0.5610	0.6012	0.2483	0.5878	
50	65.45	**0.0001*****	**0.0001*****	**0.0001*****	**0.0001*****	**0.0001*****	**0.0012****

BODIPY

	Means	0	1	2	5	10	20
0	23.17						
1	18.43	0.9998					
2	17.73	0.9996	1.0000				
5	16.63	0.9990	1.0000	1.0000			
10	11.87	0.9813	0.9999	0.9995	0.9998		
20	34.63	0.9799	0.9032	0.8852	0.8536	0.6797	
50	80.97	**0.0149***	**0.0081****	**0.0074****	**0.0064****	**0.0035****	0.0641

H_2DCFDA

	Means	0	1	2	5	10	20
0	1.2						
1	0.85	1.0000					
2	1.05	1.0000	1.0000				
5	16.8	0.5322	0.4784	0.5105			
10	27.53	0.0631	0.5250	0.0587	0.8499		
20	30.5	**0.0299***	**0.0246***	**0.0277***	0.6567	0.9998	
50	99.4	**0.0001*****	**0.0001*****	**0.0001*****	**0.0001*****	**0.0001*****	**0.0001*****

Supplementary Table 3. Statistical significance testing for data presented in **Figure 8**. ***p<0.001; **p<0.01; *p<0.05. The left column indicates concentrations of TiO_2NPs in mg/L. Mean elasticity in MPa, mean k_{cell} in N/m and mean indentation δ in nm.

JW3606

Elasticity *E*

	Means	0	1	2	5	10
**0**	3.316					
**1**	4.147	0.0851				
**2**	5.391	**0.0206***	0.2442			
**5**	4.224	0.0528	0.3993	0.3308		
**10**	2.526	0.3039	**0.0255***	**0.0041****	**0.0137***	
**20**	7.193	**0.0001*****	**0.0093****	**0.0485***	**0.0180***	**0.0000*****

Cell stiffness k_{cell}

	Means	0	1	2	5	10
**0**	0.106					
**1**	0.128	0.0768				
**2**	0.143	**0.0422***	0.3783			
**5**	0.123	0.1753	0.3048	0.2060		
**10**	0.109	0.4859	0.0651	**0.0341***	0.1580	
**20**	0.168	**0.0003*****	**0.0206***	**0.0416***	**0.0053****	**0.0002*****

Indentation δ

	Means	0	1	2	5	10
**0**	40.1					
**1**	38.05	0.2879				
**2**	33.15	**0.0446***	0.1190			
**5**	35.29	0.1293	0.2776	0.2776		
**10**	44.89	0.1035	**0.0313***	**0.0013****	**0.0075****	
**20**	25.27	**0.0003*****	**0.0016****	**0.0351***	**0.0087****	**0.0000*****

JW3596

Elasticity *E*

	Means	0	1	2	5	10
**0**	2.329					
**1**	1.687	0.1460				
**2**	1.588	0.0874	0.3807			
**5**	2.142	0.3538	0.2487	0.1630		
**10**	4.907	0.0692	**0.0056****	**0.0023****	**0.0316***	
**20**	6.364	**0.0048****	**0.0001*****	**0.0000*****	**0.0015****	0.1341

Cell stiffness k_{cell}

	Means	0	1	2	5	10
**0**	0.103					
**1**	0.08	0.1253				
**2**	0.074	0.0651	0.3577			
**5**	0.078	0.1024	0.4370	0.4232		
**10**	0.134	0.1536	**0.0150***	**0.0056****	**0.0121***	
**20**	0.152	0.0266	**0.0010****	**0.0003*****	**0.0009*****	0.1810

Indentation δ

	Means	0	1	2	5	10
**0**	48.82					
**1**	53.16	0.2274				
**2**	62.7	0.1075	0.3262			
**5**	50.55	0.4709	0.2492	0.1216		
**10**	35.22	**0.0370***	**0.0067****	**0.0012****	**0.0315***	
**20**	28.14	**0.0020****	**0.0002**	**0.0000*****	**0.0016****	0.1370

Supplementary Table 4. Statistical significance testing for data presented in **Figure 9**. ****p*<0.001; ***p*<0.01; **p*<0.05. The left column indicates concentrations of TiO_2NPs in mg/L.

JW3606

ompF

	Means	0	1	2	5	10
**0**	5.19					
**1**	4.25	0.285				
**2**	4.14	0.186	1.000			
**5**	4.63	0.774	0.946	0.853		
**10**	5.25	1.000	0.224	0.142	0.686	
**20**	6.40	0.093	**0.001*** **	**0.0005*** **	**0.006** **	0.124

osmB

	Means	0	1	2	5	10
**0**	9.88					
**1**	9.33	0.760				
**2**	9.77	1.000	0.888			
**5**	9.89	1.000	0.745	1.000		
**10**	8.65	0.072	0.577	0.119	0.068	
**20**	7.81	**0.001** **	**0.018***	**0.002** **	**0.001*** **	0.361

osmC

	Means	0	1	2	5	10
**0**	3.25					
**1**	3.02	0.995				
**2**	3.12	1.000	1.000			
**5**	3.89	0.711	0.411	0.535		
**10**	3.54	0.986	0.853	0.932	0.966	
**20**	2.78	0.886	0.993	0.968	0.176	0.543

otsB

	Means	0	1	2	5	10
**0**	3.89					
**1**	3.47	0.777				
**2**	3.8	1.000	0.898			
**5**	4.49	0.455	0.051	0.316		
**10**	4.05	0.996	0.487	0.970	0.747	
**20**	2.25	**0.001*** **	**0.015***	**0.002** **	**0.001*** **	**0.001*** **

oppA

	Means	0	1	2	5	10
**0**	6.75					
**1**	6.23	0.718				
**2**	6.37	0.898	0.999			
**5**	6.16	0.615	1.000	0.993		
**10**	5.14	**0.004*** **	0.080	0.038	0.111	
**20**	3.41	**0.0001*** **	**0.0001*** **	**0.0001*** **	**0.0001*** **	**0.002** **

lpxC

	Means	0	1	2	5	10
**0**	6.59					
**1**	6.30	0.937				
**2**	6.45	0.997	0.997			
**5**	6.52	1.000	0.981	1.000		
**10**	5.65	0.069	0.333	0.160	0.108	
**20**	5.33	**0.009** **	0.056	**0.022***	**0.014***	0.904

JW3596

ompF

	Means	0	1	2	5	10
**0**	6.95					
**1**	7.26	0.996				
**2**	7.12	1.000	1.000			
**5**	7.74	0.815	0.970	0.920		
**10**	9.01	0.065	0.139	0.100	0.409	
**20**	9.46	**0.02***	**0.045***	**0.032***	0.154	0.979

osmB

	Means	0	1	2	5	10
**0**	10.77					
**1**	10.69	1.000				
**2**	10.12	0.928	0.957			
**5**	10.70	1.000	1.000	0.952		
**10**	10.08	0.912	0.945	1.000	0.939	
**20**	9.17	0.258	0.303	0.738	0.294	0.766

osmC

	Means	0	1	2	5	10
**0**	4.49					
**1**	4.38	1.000				
**2**	4.00	0.945	0.982			
**5**	4.13	0.986	0.998	1.000		
**10**	3.68	0.697	0.805	0.991	0.959	
**20**	3.32	0.347	0.446	0.816	0.691	0.984

otsB

	Means	0	1	2	5	10
**0**	9.19					
**1**	9.09	1.000				
**2**	8.81	0.997	1.000			
**5**	8.92	0.998	1.000	1.000		
**10**	8.54	0.909	0.954	0.993	0.991	
**20**	8.03	0.521	0.613	0.781	0.760	0.969

oppA

	Means	0	1	2	5	10
**0**	5.48					
**1**	5.44	1.000				
**2**	5.14	1.000	1.000			
**5**	5.17	1.000	1.000	1.000		
**10**	5.38	1.000	1.000	1.000	1.000	
**20**	5.63	1.000	1.000	0.996	0.997	1.000

lpxC

	Means	0	1	2	5	10
**0**	8.74					
**1**	9.98	0.998				
**2**	8.62	1.000	0.985			
**5**	8.93	1.000	1.000	0.993		
**10**	8.65	1.000	0.990	1.000	0.996	
**20**	8.12	0.873	0.653	0.942	0.707	0.927

Supplementary Table 5. Statistical significance testing for data presented in **Supplementary Figure 3**. ****p*<0.001; ***p*<0.01; **p*<0.05. The left column indicates concentrations of TiO_2NPs in mg/L.

JW3606

sodA

	Means	0	1	2	5	10
**0**	3.36					
**1**	2.83	0.856				
**2**	2.84	0.869	1.000			
**5**	3.66	0.985	0.491	0.509		
**10**	4.32	0.341	**0.046***	**0.049***	0.713	
**20**	3.62	0.991	0.535	0.554	1.000	0.669

sodB

	Means	0	1	2	5	10
**0**	0.05					
**1**	-0.11	0.989				
**2**	-0.31	0.722	0.965			
**5**	0.15	0.998	0.898	0.470		
**10**	0.91	**0.028***	**0.008****	**0.001****	**0.066***	
**20**	1.93	**0.0001*****	**0.0001*****	**0.0001*****	**0.0001*****	**0.008****

sodC

	Means	0	1	2	5	10
**0**	2.92					
**1**	2.38	0.580				
**2**	2.60	0.919	0.983			
**5**	3.10	0.993	0.287	0.653		
**10**	2.09	0.168	0.947	0.639	0.061	
**20**	1.67	**0.014***	0.298	0.096	**0.004****	0.787

katG

	Means	0	1	2	5	10
**0**	3.09					
**1**	2.68	0.974				
**2**	2.26	0.675	0.972			
**5**	2.46	0.859	0.998	0.999		
**10**	2.15	0.557	0.928	1.000	0.993	
**20**	1.85	0.275	0.677	0.975	0.881	0.994

ahpC

	Means	0	1	2	5	10
**0**	-0.63					
**1**	-1.27	0.220				
**2**	-1.24	0.270	1.000			
**5**	-0.92	0.880	0.780	0.850		
**10**	-0.17	0.550	**0.008****	0.010	0.108	
**20**	0.08	0.140	**0.001****	**0.001****	0.017	0.934

JW3596

sodA

	Means	0	1	2	5	10
**0**	3.49					
**1**	3.17	0.932				
**2**	3.08	0.822	1.000			
**5**	2.84	0.434	0.910	0.977		
**10**	2.88	0.497	0.945	0.990	1.000	
**20**	2.92	0.563	0.969	0.996	1.000	1.000

sodB

	Means	0	1	2	5	10
**0**	-2.74					
**1**	-2.35	0.975				
**2**	-2.59	1.000	0.997			
**5**	-2.08	0.821	0.995	0.928		
**10**	-2.12	0.854	0.998	0.948	1.000	
**20**	-1.76	0.488	0.872	0.645	0.988	0.981

sodC

	Means	0	1	2	5	10
**0**	6.56					
**1**	6.43	1.000				
**2**	5.82	0.773	0.879			
**5**	6.30	0.997	1.000	0.951		
**10**	5.82	0.773	0.879	1.000	0.951	
**20**	5.06	0.157	0.221	0.758	0.306	0.758

katG

	Means	0	1	2	5	10
**0**	0.98					
**1**	1.04	1.000				
**2**	1.05	1.000	1.000			
**5**	0.82	1.000	0.998	0.997		
**10**	0.64	0.984	0.970	0.966	1.000	
**20**	0.83	1.000	0.998	0.998	1.000	0.999

ahpC

	Means	0	1	2	5	10
**0**	-2.64					
**1**	-1.72	0.718				
**2**	-1.54	0.560	1.000			
**5**	-2.07	0.946	0.993	0.958		
**10**	-1.54	0.558	1.000	1.000	0.957	
**20**	-0.79	0.115	0.707	0.847	0.406	0.850
