# Supplementary material for: Osmotic stress and vesiculation as key mechanisms controlling bacterial sensitivity and resistance to TiO2 nanoparticles
Source: Commun Biol. 2021 Jun 3;4:678. doi: 10.1038/s42003-021-02213-y (PMC8175758; doi:10.1038/s42003-021-02213-y)
Supplement: Supplementary file 2 — Description of Supplementary Files [file 42003_2021_2213_MOESM2_ESM.pdf]

Description of Additional Supplementary Files

File name: Supplementary Data

Description:

• Supplementary Data 1. This excel file details the source data associated with Figure 1b.

• Supplementary Data 2. This excel file details the source data associated with Figure 2.

• Supplementary Data 3. This excel file details the source data associated with Figure 3, which includes the data pertaining to the reported electrophoretic mobility distributions of bacteria and membrane vesicles, and to the n maxima values for peak amplitudes adopted for the evaluation of the distribution of most frequent mean electrophoretic mobility.

• Supplementary Data 4. This excel file details the source data associated with Figures 4a-(ii) and Figure 4b-(ii), which includes the data pertaining to the reported size distributions of the membrane vesicles (MV) obtained by AFM.

• Supplementary Data 5. This excel file details the source data associated with Figures 4a-(iii) and Figure 4b-(iii), which includes the data pertaining to the reported size distributions of the membrane vesicles obtained by dynamic light scattering and to the n maxima values for peak amplitudes adopted for the evaluation of the distribution of most frequent mean vesicle size.

• Supplementary Data 6. This excel file details the source data associated with Figures 5b,c,d.

• Supplementary Data 7. This excel file details the source data associated with Figure 6b-(i). The file reports the 256•256 matrix of dimensionless elasticity values of JW3606 (hep+) for the various TiO2NP concentrations tested in the work.

• Supplementary Data 8. This excel file details the source data associated with Figure 6b-(ii). The file reports the 256•256 matrix of dimensionless stiffness of JW3606 (hep+) for the various TiO2NP concentrations tested in the work.

• Supplementary Data 9. This excel file details the source data associated with Figure 6b-(iii). The file reports the 256•256 matrix of dimensionless indentation • (defined in the main text) of JW3606 (hep+) for the various TiO2NP concentrations tested in the work.

• Supplementary Data 10. This excel file details the source data associated with Figure 6c-(i).

• Supplementary Data 11. This excel file details the source data associated with Figure 6c-(ii).

• Supplementary Data 12. This excel file details the source data associated with Figure 6c-(iii).

• Supplementary Data 13. This excel file details the source data associated with Figure 7b-(i). The file reports the 256•256 matrix of dimensionless elasticity values of JW3596 (hep-) for the various TiO2NP concentrations tested in the work.

• Supplementary Data 14. This excel file details the source data associated with Figure 7b-(ii). The file reports the 256•256 matrix of dimensionless stiffness of JW3596 (hep-) for the various TiO2NP concentrations tested in the work.

• Supplementary Data 15. This excel file details the source data associated with Figure 7b-(iii). The file reports the 256•256 matrix of dimensionless indentation • (defined in the main text) of JW3596 (hep-) for the various TiO2NP concentrations tested in the work.

• Supplementary Data 16. This excel file details the source data associated with Figure 7c-(i).

• Supplementary Data 17. This excel file details the source data associated with Figure 7c-(ii).

• Supplementary Data 18. This excel file details the source data associated with Figure 7c-(iii).

• Supplementary Data 19. This excel file details the source data associated with Figure 8a-(i) and Figure 8b-(i).

• Supplementary Data 20. This excel file details the source data associated with Figure 8a-(ii) and Figure 8b-(ii).

• Supplementary Data 21. This excel file details the source data associated with Figure 8a-(iii) and Figure 8b-(iii).

• Supplementary Data 22. This excel file details the source data associated with Figure 9.
