# Supplementary material for: Osmotic stress and vesiculation as key mechanisms controlling bacterial sensitivity and resistance to TiO2 nanoparticles
Source: Commun Biol. 2021 Jun 3;4:678. doi: 10.1038/s42003-021-02213-y (PMC8175758; doi:10.1038/s42003-021-02213-y)
Supplement: Supplementary file 4 — Reporting Summary [file 42003_2021_2213_MOESM4_ESM.pdf]

nature research

Corresponding author(s): Duval Jerome F.L.

Last updated by author(s): Apr 19, 2021

Reporting Summary

Nature Research wishes to improve the reproducibility of the work that we publish. This form provides structure for consistency and transparency in reporting. For further information on Nature Research policies, see our Editorial Policies and the Editorial Policy Checklist.

Statistics

For all statistical analyses, confirm that the following items are present in the figure legend, table legend, main text, or Methods section.

n/a	Confirmed	
☐	☒	The exact sample size (*n*) for each experimental group/condition, given as a discrete number and unit of measurement
☐	☒	A statement on whether measurements were taken from distinct samples or whether the same sample was measured repeatedly
☐	☒	The statistical test(s) used AND whether they are one- or two-sided<br>*Only common tests should be described solely by name; describe more complex techniques in the Methods section.*
☒	☐	A description of all covariates tested
☐	☒	A description of any assumptions or corrections, such as tests of normality and adjustment for multiple comparisons
☐	☒	A full description of the statistical parameters including central tendency (e.g. means) or other basic estimates (e.g. regression coefficient) AND variation (e.g. standard deviation) or associated estimates of uncertainty (e.g. confidence intervals)
☒	☐	For null hypothesis testing, the test statistic (e.g. *F*, *t*, *r*) with confidence intervals, effect sizes, degrees of freedom and *P* value noted<br>*Give P values as exact values whenever suitable.*
☒	☐	For Bayesian analysis, information on the choice of priors and Markov chain Monte Carlo settings
☒	☐	For hierarchical and complex designs, identification of the appropriate level for tests and full reporting of outcomes
☒	☐	Estimates of effect sizes (e.g. Cohen's *d*, Pearson's *r*), indicating how they were calculated

Our web collection on statistics for biologists contains articles on many of the points above.

Software and code

Policy information about availability of computer code

Data collection	We did not use any open source, custom or commercial software to collect the data in this study.
Data analysis	Interpretation of nanomechanical AFM data were performed by means of a homemade code we have already described in a previous publication (Offroy, M., Razafitianamaharavo, A., Beaussart, A., Pagnout, C. & Duval, J. F. L. (2020) RSC Advances 10, 19258-19275).

For manuscripts utilizing custom algorithms or software that are central to the research but not yet described in published literature, software must be made available to editors and reviewers. We strongly encourage code deposition in a community repository (e.g. GitHub). See the Nature Research guidelines for submitting code & software for further information.

Data

Policy information about availability of data

All manuscripts must include a data availability statement. This statement should provide the following information, where applicable:

- Accession codes, unique identifiers, or web links for publicly available datasets
- A list of figures that have associated raw data
- A description of any restrictions on data availability

The authors declare that the data supporting the findings of this study are available within the paper and its supplementary information file.

Field-specific reporting

Life sciences study design

All studies must disclose on these points even when the disclosure is negative.

Sample size	Given the objective of our study, the sample size of concern primarily refers to the bacteria to TiO2NPs concentrations ratio. The later was varied by fixing the amount of cells in solution upon proper adjustment of the optical density (specified in the text) and by varying the solution concentration of TiO2NPs over the 0 to 50 mg/L range (also specified). These conditions cover the whole spectrum of possible scenarios, from the absence of TiO2NPs action, to reduced changes in cell physiology and cell death.
Data exclusions	There were no data excluded from the analysis.
Replication	To verify the reproducibility of the data obtained by flow cytometry, electrokinetics, light scattering, targeted transcriptomics and atomic force microscopy, experiments were iterated N times for each type of experiments and N is systematically specified in the manuscript (we further precised whether replicates concerned a given bacterial culture or were measured by means of different bacterial cultures). The value of N we considered for each type of experiments typically corresponds to that reported in literature. Of special interest are the AFM data collected on N=8 individual cells per examined condition, which corresponds to what is commonly reported in literature. The discussion section provides a rationale for the consistency of the cells reponse to TiO2 nanoparticles as derived by interpretation of the data set collected from the gene to population scale, which further solidly supports the relevance of the measurements/replicates collected at the various spatial scales of interest.
Randomization	Randomization was not relevant in our study: we have worked on two well-identified bacterial strains (deep-rough E. coli mutants) featuring different surface LPS compositions; the study aimed at deciphering, on a mechanistic basis, the processes that drove the toxic action modes of TiO2 nanoparticles on these two mutants and the role played by their respective LPS surface structures in mitigating or not nanoparticles toxicity.
Blinding	Blinding was not relevant in our study: we considered two well-known bacterial strains differing in terms of surface LPS compositions, and we measured and interpreted on a mechanistic basis their response to TiO2 nanoparticles (whose solution concentration was varied) via a unique combination of multi-scale techniques (from the gene, single cell to population scale) including targeted transcriptomics, multiparametric atomic force microscopy, cell electrokinetics and fluorescence-based assays.

Reporting for specific materials, systems and methods

We require information from authors about some types of materials, experimental systems and methods used in many studies. Here, indicate whether each material, system or method listed is relevant to your study. If you are not sure if a list item applies to your research, read the appropriate section before selecting a response.

Materials & experimental systems

n/a	Involved in the study	
☒	☐	Antibodies
☒	☐	Eukaryotic cell lines
☒	☐	Palaeontology and archaeology
☒	☐	Animals and other organisms
☒	☐	Human research participants
☒	☐	Clinical data
☒	☐	Dual use research of concern

Methods

n/a	Involved in the study	
☒	☐	ChIP-seq
☐	☒	Flow cytometry
☒	☐	MRI-based neuroimaging

Flow Cytometry

Plots

Confirm that:

☒ The axis labels state the marker and fluorochrome used (e.g. CD4-FITC).

☒ The axis scales are clearly visible. Include numbers along axes only for bottom left plot of group (a 'group' is an analysis of identical markers).

☒ All plots are contour plots with outliers or pseudocolor plots.

☒ A numerical value for number of cells or percentage (with statistics) is provided.

Methodology

Sample preparation	After 20 h cell exposure to TiO2NPs, samples were collected and diluted 1:50 in a KNO3 solution containing the different fluorescent probes prior to flow cytometry analyses. For each probe selected, we provide in the Methods section the supplier, the labeling time and the final concentration we used .
Instrument	Samples were analysed on a Flow cytometer BD Accuri C6 equipped with a 488 nm laser. Filters used were FL1 (530 nm) and

	FL3 (LP 670 nm). The threshold was set to 15000 of FSC. For detection of membrane vesicles (MVs), the trigger was set at 1000 on FL3 and 100 on FSC. This procedure is described in the Methods section.
Software	Data analysis was performed with using the Accuri software from BD Biosciences.
Cell population abundance	The percentage of cells was determined by measuring the number of Syto9 positive events in the pool of total events detected. The absence of non specific labeling of TiO2NPs aggregates was further controlled.
Gating strategy	For the experiments targeting the impact of TiO2NPs on bacteria, no FSC/SSC gates were used as the analyzed samples only contained the labelled bacteria of interest. For the experiments pertaining to MVs detection/analysis (Fig5A), a gate on "FM4-64 positive" particules was set to separate MVs from bacteria in the FSC signal (gate mentionned in the figure caption). The percentage of cells detected versus the number of particules was determined with using the Syto9 probe. The boundaries between "positive" and "negative" staining cells were defined with unlabeled cells -in the presence and absence of nanoparticles (negative control)- for the membrane and nucleic acid selective dyes FM4-64 and Syto9, respectively. Concerning the probes selected for membrane depolarisation (DIBAC4(3)), membrane permeability (propidium iodide), lipid peroxidation (BODIPY) and oxidative stress (H2DCFDA), two positive controls were also used: cells heated for 10 min at 65°C and cells incubated for 20 min with 0.9 % of H2O2. Flow cytometry gating strategies adopted in this study use specific fluorescent DNA-labeling probe (Syto9) to determine the overall bacterial population and conventional bivariate gating methods. These elements are described in the Methods section and additional figure in the Supplementary Information is thus not required.

☐ Tick this box to confirm that a figure exemplifying the gating strategy is provided in the Supplementary Information.
